# Hierarchical Chunking of Sequential Memory on Neuromorphic Architecture with Reduced Synaptic Plasticity

**DOI:** 10.3389/fncom.2016.00136

**Published:** 2016-12-20

**Authors:** Guoqi Li, Lei Deng, Dong Wang, Wei Wang, Fei Zeng, Ziyang Zhang, Huanglong Li, Sen Song, Jing Pei, Luping Shi

**Affiliations:** ^1^Department of Precision Instrument, Center for Brain Inspired Computing Research, Tsinghua UniversityBeijing, China; ^2^School of Automation Science and Electric Engineering, Beihang UniversityBeijing, China; ^3^Department of Materials Science and Engineering, Tsinghua UniversityBeijing, China; ^4^School of Medicine, Tsinghua UniversityBeijing, China

**Keywords:** chunking, synaptic plasticity, sequential memory, neuromorphic engineering, memristor

## Abstract

Chunking refers to a phenomenon whereby individuals group items together when performing a memory task to improve the performance of sequential memory. In this work, we build a bio-plausible hierarchical chunking of sequential memory (HCSM) model to explain why such improvement happens. We address this issue by linking hierarchical chunking with synaptic plasticity and neuromorphic engineering. We uncover that a chunking mechanism reduces the requirements of synaptic plasticity since it allows applying synapses with narrow dynamic range and low precision to perform a memory task. We validate a hardware version of the model through simulation, based on measured memristor behavior with narrow dynamic range in neuromorphic circuits, which reveals how chunking works and what role it plays in encoding sequential memory. Our work deepens the understanding of sequential memory and enables incorporating it for the investigation of the brain-inspired computing on neuromorphic architecture.

## 1. Introduction

The word “Chunking,” a phenomenon whereby individuals group items together when performing a memory task, was initiated by (Miller, 1956). (Lindley, 1966) showed that groups produced by chunking have concept meanings to the participant. Therefore, this strategy makes it easier for an individual to maintain and recall information in memory. For example, when recalling a number sequence 01122014, if we group the numbers as 01, 12, and 2014, mnemonic meanings for each group as a day, a month and a year are created. Furthermore, studies found evidence that the firing event of a single cell is associated with a particular concept, such as personal names of Bill Clinton or Jennifer Aniston (Kreiman et al., 2000, 2001).

Psychologists believe that chunking plays as an essential role in joining the elements of a memory trace together through a particular hierarchical memory structure (Tan and Soon, 1996; Edin et al., 2009). At a time when information theory started to be applied in psychology, Miller claimed that short-term memory is not rigid but amenable to strategies (Miller, 1956) such as chunking that can expand the memory capacity (Gobet et al., 2001). According to this information, it is possible to increase short-term memory capacity by effectively recoding a large amount of low-information-content items into a smaller number of high-information-content items (Cowan, 2001; Chen and Cowan, 2005). Therefore, when chunking is evident in recall tasks, one can expect a higher proportion of correct recalls. Patients with Alzheimer's disease typically experience working memory deficits; chunking is also an effective method to improve patients' verbal working memory performance (Huntley et al., 2011).

However, to this day, the mechanism why chunking improves human memory is unclear. This is mainly due to two difficulties. Firstly, no mathematical model is applicable to describe the memory processing in human brain. Secondly, no bio-plausible validation system that allows to emulate how chunking can be merged into a proper memory model. Although researchers have a long way to go before synthetic systems can match the capability of the natural brain, there are breakthroughs in neuroscience and neuromorphic engineering studies (Mead, 1989):

1. *The discovery of the link between transient metastability and sequential memory in the brain*. Advances in non-invasive brain imaging (Gholipour et al., 2007) allow assessing the structural connectivity of the brain and corresponding evolution of the spatio-temporal activity in details. This makes the structure and dynamics of functional brain networks useful for building theoretical memory models. Among these results, one popular view is that, sequential memory, which refers to the functionality of the brain to encode and represent the temporal order of discrete elements occurring in a sequence, plays a key role in organizing the brain memory. The metastable state (Rabinovich et al., 2008, 2014; Mante et al., 2013; Tognoli and Kelso, 2014) is a significant feature of sequential memory. Experimental and modeling studies suggest that most of the sequential memories are the result of transient activities of large-scale brain networks in the presence of noise (Rabinovich et al., 2008; Maass, 2014).
2. *The discovery of the bridge between the synapse and the memristor*. A synapse is a functional unit of the brain, which permits a neuron to pass an electrical or chemical signal to another neuron. In the last few years, it is believed that a synapse bears striking resemblance to a two-terminal electrical device termed as “memristor” (memory + resistor) (Chua, 1971; Strukov et al., 2008; Kim et al., 2012). The memristor resistive states can be modified by controlling the voltage applied across its terminals or the current flowing through it, which makes it promising to emulate the biological synapse (Jo et al., 2010; Chang et al., 2011; Kuzum et al., 2011; Yu et al., 2011; Alibart et al., 2012; Jackson et al., 2013; Kuzum et al., 2013; He et al., 2014). Clearly, advancements in memristor technology are establishing entirely new fashions in brain-inspired chip design.

**Figure 1:**
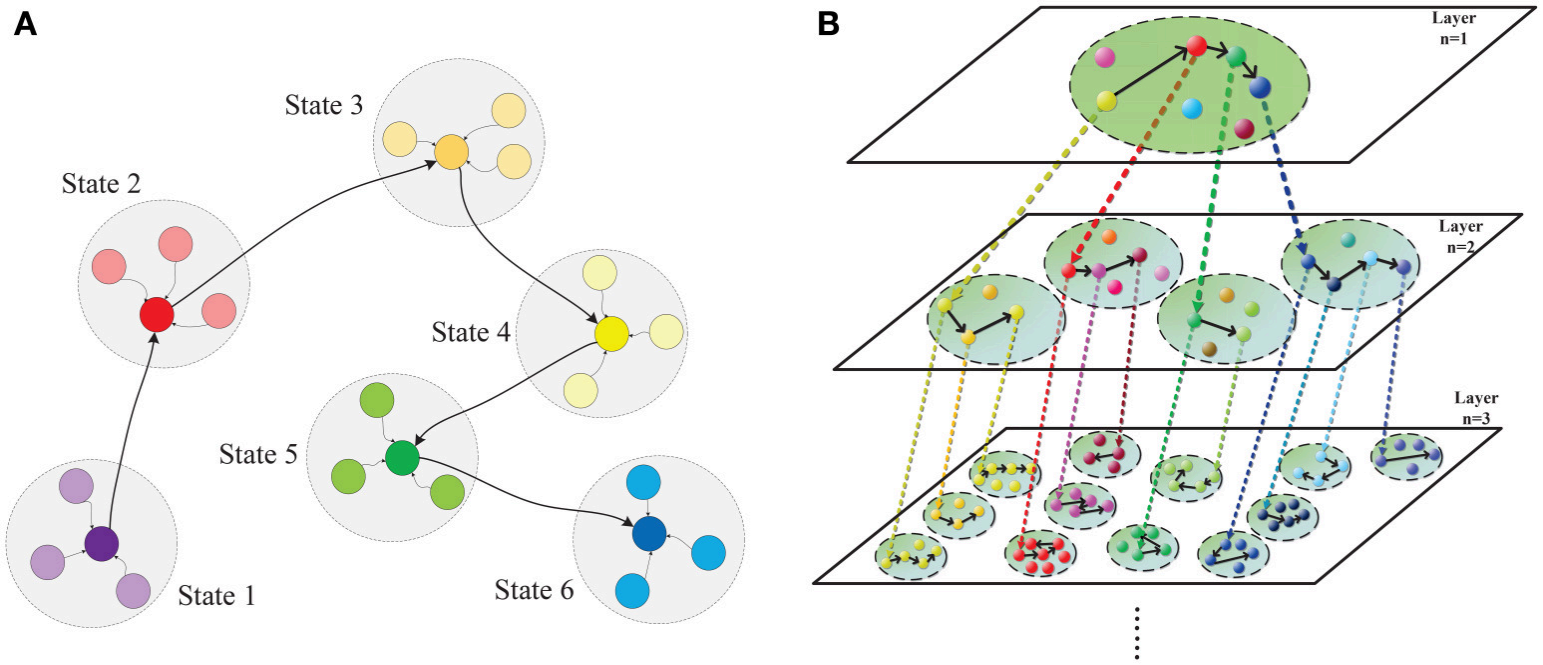
**Hierarchical chunking of sequential memory (HCSM) model. (A)** The metastable states within a chunk, within which neurons are all-to-all connected. Each colored node denotes a temporal winner neuron in the chunk, which is a metastable state switching from one to another in the sequential memory trace. The shadowed circle encompassing each metastable state represents the domain of attraction for the state. **(B)** An illustrative diagram of the proposed hierarchical chunking sequential memory model. The chunk indexed by (*n, m*) denotes the *m*th chunk in layer *n*. A neuron representing a particular concept in a chunk (termed as PC) in layer *n* may activate a chunk (termed as CC) in layer *n* + 11 that is connected to the PC neuron through a dotted arrow. In a memory recall, as the metastable neuron switches from one to another in a PC, the corresponding CCs is activated in a sequential order.

Based on above breakthroughs, we set out to investigate why chunking improves sequential memory performance. To achieve this, we build a bio-plausible hierarchical chunking of sequential memory (HCSM) model shown in Figure 1 using memristors as synapses. More specifically, our works are summarized as follows. Firstly, a HCSM model consisting multilayered neural networks is proposed. Each layer is divided into different chunks of neurons. Within each chunk, neurons are all-to-all connected (**Figure 3**); while chunks in different layers might be correlated through an activation signal denoted by the dotted arrows in Figure 1. In particular, a chunk in the upper layer and its connected ones in the adjacent lower layer are termed as parent chunk (PC) and child chunks (CCs), respectively. A winner neuron in a PC activates its connected child chunk (CC) to form a hierarchical structure (Figure S4). The winnerless competition (WLC) (Rabinovich et al., 2001) principle is applied between neurons, i.e., the winner is temporary or “metastable” because it switches from one neuron to another. The HCSM model selects the necessary metastable states and link them together to form a sequential memory through the hierarchical organization. When a recall cue is given, the model presents a memory trace containing temporary winner neurons among different chunks. The trace reflects the sequential memory recall. Secondly, to emulate the synapse with ideal synaptic plasticity, we use iron oxide (He et al., 2014) as the memristor resistive layer. A memristor with a typical sandwich structure, 0.25 μ*m*^2^−*size TiW*/*Pt*/*FeO*_*x*_/*Pt*/*TiW*, is fabricated, as shown in Figure S1. The well-known I-V hysteresis loops of memristor (Chua, 2011) under applied triangle-wave-shape DC voltage sweeps are observed. The conductance of this memristor can be monotonically and consecutively modulated among the intermediate states, which is crucial for the synaptic plasticity emulation. Lastly, we provide a neuromorphic chip implementation (**Figure 3**, Figures S2–S4) in which the memristor crossbar is used for emulating the synapse matrix of each chunk in the proposed HCSM model, and a scheme for encoding the sequential memory is presented. The key to encode memory in a bio-neural network is to exploit its ability of changing the synaptic weights (Zeng et al., 2001), which is also known as synaptic plasticity. In fact, synaptic plasticity is widely believed to be essential for creating the memory and learning ability of the brain (Hebb, 1949; Bi and Poo, 1998; Song et al., 2000; Han et al., 2011; Ramanathan et al., 2012; Carrillo-Reid et al., 2015).

With the HCSM model, the chunking mechanism can be linked to the synaptic plasticity. Usually, the dynamic range of the synapse, i.e., a memristor when considering a neuromorphic chip, is required to be much wider if the same length of sequential memory is encoded without chunking. By contrast, we observed that only a narrower dynamic range and imprecise state of the synaptic weight is required to encode a sequential memory with chunking mechanism. Thus, it is shown that chunking improves sequential memory by reducing the requirements of synapse plasticity in memory encoding. Our work reveals how chunking works and what role it plays in encoding sequential memory.

## 2. Materials and methods

**Figure 2:**
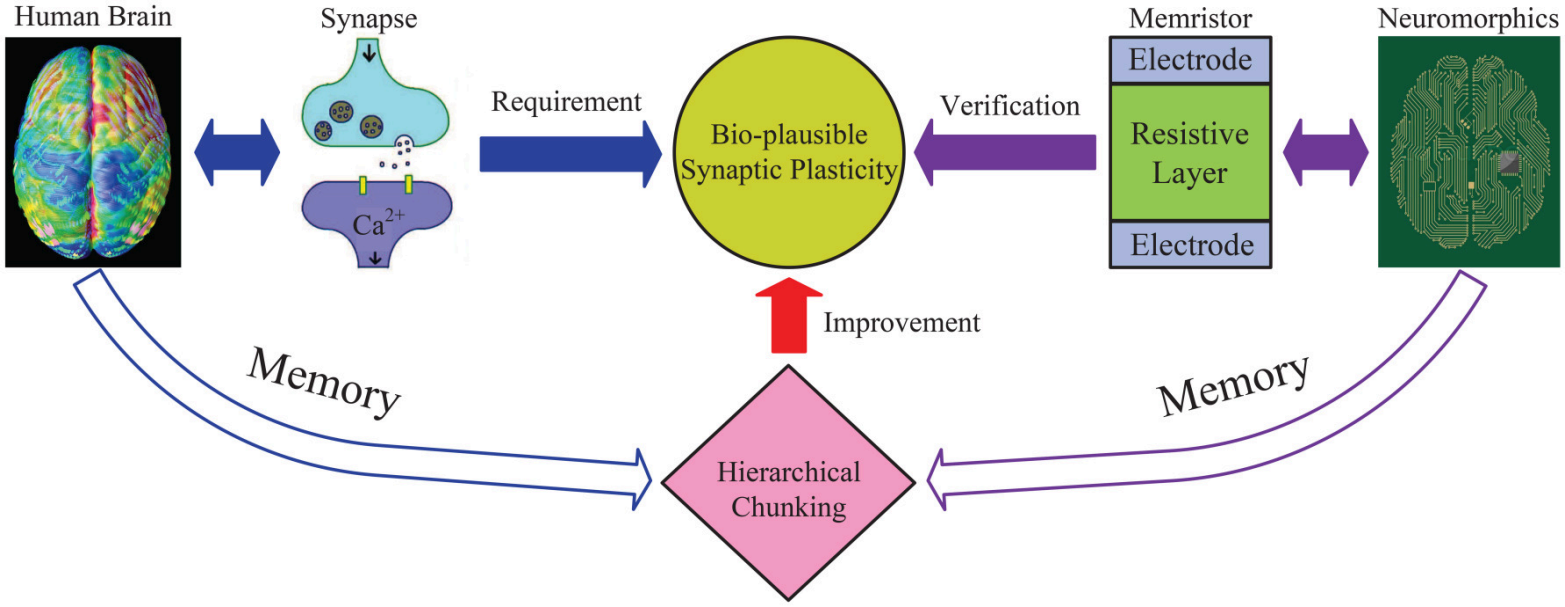
**An illustrative diagram of the main idea in this work**. General neural networks without chunking require the synapse to have wide dynamic range, especially to memorize a long sequence. While the proposed hierarchical chunking mechanism greatly improves memory performance under a lower requirement for synaptic plasticity, i.e., only requires a narrow dynamic range and not a very precise state, which seems bio-plausible. A neuromorphic architecture is designed based on memristor devices with narrow dynamic range to successfully perform the sequential memory simulation. In this way, the reduced “synaptic plasticity” provided by chunking model bridges the brain memory dynamics and neuromorphic system.

As illustrated in Figure 2, this work explains why hierarchical chunking mechanism helps improve the memory performance and provides a promising solution to successfully realize memory dynamics in neuromorphic circuits. Through the reduced “synaptic plasticity” provided by the chunking mechanism, i.e., narrow dynamic range and not so precise state, we establish a bridge between the brain memory dynamics and the neuromorphic system.

### 2.1. Synapse and memristor

The molecular nature of the synaptic plasticity has been mathematically examined to have identical calcium-dependent dynamics, where the synaptic weight is described by a linear equation as follows (Shouval et al., 2002):

(1) $$\begin{array}{l} \frac{dW_{i}}{dt}=\frac{1}{\tau ({\left[Ca^{2+}\right]}_{i})}\left(\Omega ({\left[Ca^{2+}\right]}_{i})-W_{i}\right), \end{array}$$

where *W*_*i*_ is the synaptic weight of the *i*-th input axon. τ is a time constant with respect to the insertion and removal rates of neurotransmitter receptors, which is a function dependent on the concentration of calcium [*Ca*^2+^]. Ω is another function of [*Ca*^2+^] that depends linearly on the number of receptors on the membrane of the neuron. Equation (1) implies that the present synaptic weight between neurons is dependent on the historical weight indirectly, and it can be adjusted by changing Ω([*Ca*^2+^]).

To mimic the biological synapse, it is critical to build an artificial synaptic device to emulate its plastic behavior. Fortunately, the memristor (Strukov et al., 2008) was successfully developed and found to bear striking resemblance to the synapse in neural networks. The fundamental characteristic of a memristor is that its present resistance is dependent on its historical resistances. The resistance of a memristor can be adjusted by changing the applied voltage or current, which controls the transport of charge carriers in the nanoscale device. In this work, iron oxide is adopted as the memristor resistive layer. As shown Figure S1 in the Supplementary Information, a typical memristor of sandwich structure with $0.25\mu m^{2}-sizeTiW/Pt/FeO_{x}/Pt/TiW$ is fabricated.

However, as shown in (Kuzum et al., 2013), the dynamic range of memristor conductance to effectively emulate a synapse is often relatively narrow. For instance, the iron oxide memristor fabricated in this work is an ideal synaptic device due to its monotonous and consecutive state distribution. Note that the resistive ratio of the maximum conductance to the minimum conductance reflects the dynamic range of synaptics weight. As seen in Figure S1C, the ratio is only about 3 ~ 4. This is consistent with a narrow distribution of biological synaptic weights that generally follows a lognormal distribution (Song et al., 2005; Teramae and Fukai, 2014). With the proposed HCSM model, it will be shown later that the neuromorphic system also works well since HCSM reduces the requirements on the synaptic plasticity.

### 2.2. Hierarchical chunking of sequential memory (HCSM) model

**Figure 3:**
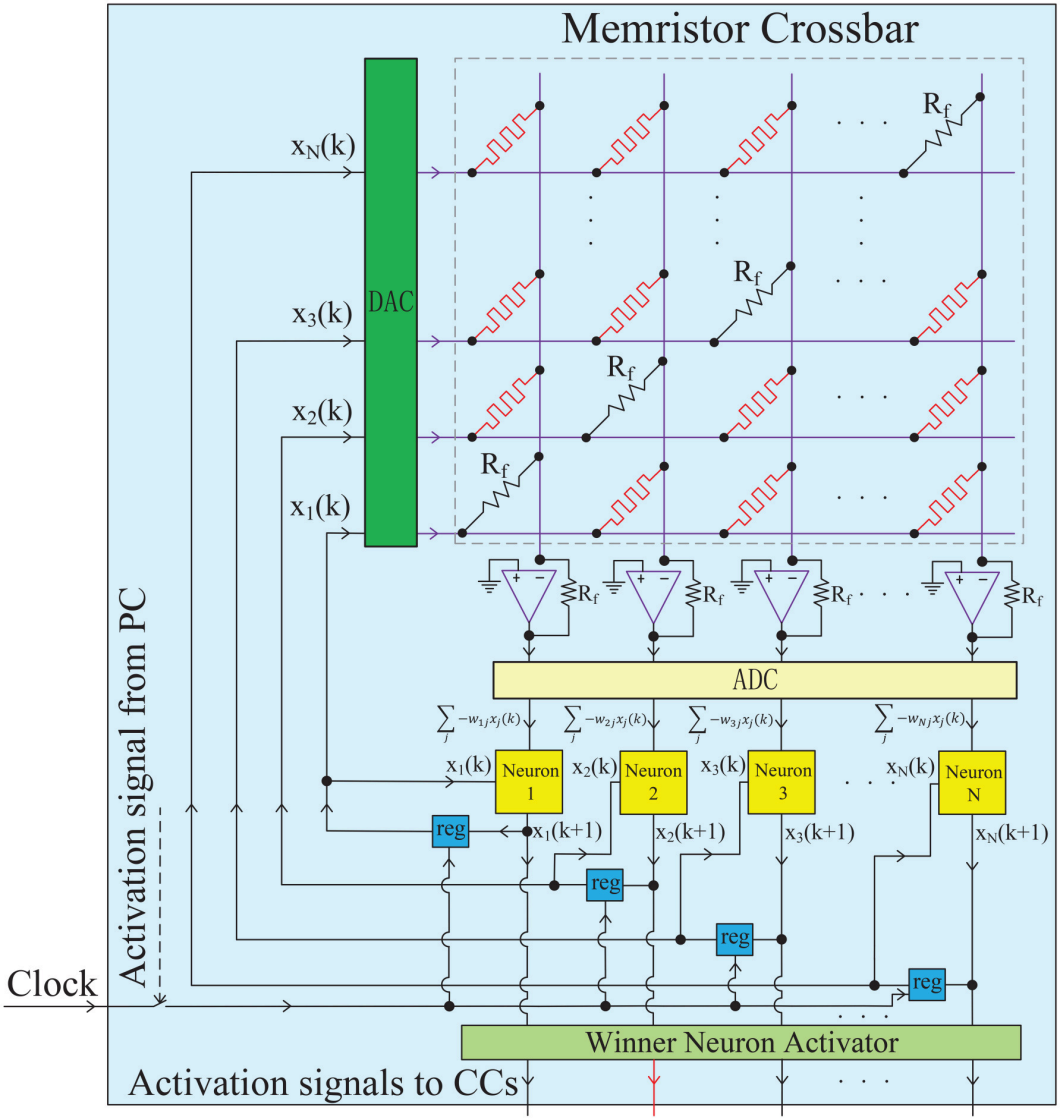
**Hardware implementation for a single chunk**. The chunk, a PC or a CC, is mainly constructed by an analog memristor crossbar circuit and digital neurons. The memristor crossbar completes a VMM operation in one step, and the digital neurons run the neuronal dynamics described in Equation (6). The diagonal elements of the crossbar are non-plastic, with the same value as *R*_*f*_ to satisfy *w*_*ii*_ = 1. The DACs and ADCs are required to convert the signal format between analog circuits and digital circuits. The iterative timing sequence *k* → *k* + 1 is governed by the clock signal. The winner neuron activator in each chunk (PC) is used to determine the winner neuron at each time step and transmit an excitatory signal to the corresponding connected CC block. When a CC receives an excitatory activation signal from a winner neuron in the PC, the clock will be triggered and the iterative neuronal dynamics in this CC starts to form a pre-defined memory trace.

We propose a hierarchical chunking of sequential memory (HCSM) model shown in Figure 1, which consists of multi-layered networks. Each dashed circle indexed by a unique tuple (*n, m*) represents the *m*th chunk in the *n*th layer. Within each chunk, neurons are all-to-all connected (Figure 3). It can be seen that in each layer *n*, each neuron is connected to a specific sub-chunk in layer *n* + 11 to form a hierarchical structure (Figure S4), through an activation signal denoted by the dotted arrows in Figure 1. Thus we refer a chunk in layer *n* and its connected chunks in layer *n* + 11 as a parent chunk (PC) and child chunks (CCs), respectively. Clearly, a chunk in layer *n* is a CC, with respect to its PC in layer *n* − 1, and also a PC with respect to its CCs in layer *n* + 11. In other words, PCs and CCs only represent relative relationships between connected chunks from two successive layers.

The winnerless competition principle (WLC) (Rabinovich et al., 2001) among neurons in each network is described by the generalized Lotka-Volterra model in (Bick, 2009). The neuron preserving the maximum activity is a winner neuron. Here “winnerless” implies a winner is only metastable and it will switch from one neuron to another in a sequential memory trace as shown in Figure 1A. As each temporary winner neuron in a PC chunk will activate its connected CC, competition exists among different chunks in the same layer. When a recall cue is given, the HCSM model presents a memory trace containing all temporary winner neurons as shown in Figure 1B. In order to apply the WLC principle to the model of hierarchical architecture in this work, a time constant (τ > 0) is introduced to reflect the dynamic evolving rate in the generalized Lotka-Volterra model. The WLC in a chunk indexed by (*n, m*) is then described by the following dynamic equation:

(2) $$\begin{array}{l} {\dot{x}}_{i}^{\left(n,m\right)}=\tau^{\left(n,m\right)}\cdot x_{i}^{\left(n,m\right)}(\sigma_{i}^{\left(n,m\right)}-\sum_{j\;=\;1}^{N_{0}^{\left(n,m\right)}}w_{ij}^{\left(n,m\right)}\cdot x_{j}^{\left(n,m\right)})+v_{i}^{\left(n,m\right)} \end{array}$$

where τ^(*n, m*)^ is the time constant that reflects the rate of activation and decay of $N_{0}^{\left(n,m\right)}$ neurons in the chunk (*n, m*), $\sigma_{i}^{\left(n,m\right)}$ is a fixed bias term that determines the equilibrium neural activity in the absence of external inputs and noise, $x_{i}^{\left(n,m\right)}\geq 0$ is the output neural activity of neuron *i*, $w_{ij}^{\left(n,m\right)}\geq 0$ for *j* ≠ *i* is the inhibitory weight (Brunel and Wang, 2001) from neuron *j* to neuron *i*, $w_{ii}^{\left(n,m\right)}=1$ for $i=1,\ldots ,N_{0}^{\left(n,m\right)}$, and $v_{i}^{\left(n,m\right)}$ is the external noise in the interval [−ε, ε] where ε is a small positive constant. Note that the weight $w_{ij}^{\left(n,m\right)}$, and $\sigma_{i}^{\left(n,m\right)}$ for all *i* and *j* are required to be encoded when performing specific task. Also, the connection between the nodes in different layers is not reflected in the dynamic Equation (2). However, it is assumed that a neuron in the PC can activate a CC chunk that is connected to the PC neuron through a activation signal denoted by dotted arrow. This implies that different chunks, for example the chunks in layer 2 of Figure 1, do not interact with each other directly in the same layer, but they indeed interact with each other in a particular way in the higher layer (layer 1). The time constant τ^(*n, m*)^ for a CC is normally smaller than that in a PC, which implies that the chunks in the CC layers possess relatively faster dynamic evolving rates. Since the HCSM model may be a deep architecture, the time constant τ has a wide range. It is known that there is indeed a wide range of time constants for the neurons in the human brain, from hundreds of milliseconds to tens of seconds (Bernacchia et al., 2011).

Note that each neuron represents a particular item in memory such as a digital number or a letter of the alphabet. The neural activity in a dynamic system (Equation 2), which is time-varying, reflects the level of activity of each neuron in a neural network. At a given time instant, the neuron of the maximum neural activity among a chunk becomes the temporal winner. The corresponding item that the winner neuron represents will be recalled.

### 2.3. Encoding scheme for the HCSM neuromorphic network

Suppose that there are *N*_0_ neurons in a chunk, and each neuron represents a particular item in the memory. Before we encode a sequence containing $\kappa \leq N_{0}$ metastable states in a chunk as described in Equation (2), the bias parameter of each neuron and the weight between two arbitrary neurons need be determined first. In this work, the bias parameter of neuron *i* in a specific chunking sequence is chosen as

(3) $$\sigma_{i}=\left\{ \begin{array}{l} F_{k}\text{, if neuron }i\text{ is the }k\text{-th term of the chunking sequence}.\\ 0\text{ otherwise.} \end{array}\right.$$

where *F*_*k*_ is the *k*-th term of the Fibonacci sequence (Dunlap, 1997) with *F*_1_ = 1 and *F*_2_ = *g*. Here *g* is the “Golden ratio” (Dunlap, 1997; Livio, 2008). The synaptic weight between neurons *i* and *j* in the same chunk is then selected as:

(4) $$w_{ij}\in \left\{ \begin{array}{l} S_{1},\text{ if neurons }i\text{ and }j\text{ are adjacent in the chunking}\\ \text{ sequence}.\;\\ S_{2}\text{ otherwise}. \end{array}\right.\;$$

with

(5) $$\begin{array}{l} S_{1}=\{x|g-\frac{1}{2}<x<g\}\\ S_{2}=\{x|g^{k\;-\;1}+1<x<+\infty \} \end{array}$$

where **k** is the length of the sequence in this chunk. A detailed motivation to the above encoding scheme associated with the existence of the metastable states in a stable sequential memory trace are provided in Theorem 1 in the supplementary information.

### 2.4. Hardware implementation

As well known, the neuromorphic engineering especially the memristive system enables the hardware implementation of neural networks with ultra-low power, small size, and high speed (Kuzum et al., 2012; Yu et al., 2013; Deng et al., 2016), which aims at the future of mobile intelligence. However, the memristor with good plasticity to emulate synapse usually suffers from a narrow dynamic range. Fortunately, the proposed HCSM model efficiently reduces the requirement on synaptic plasticity. This coincidence motivates us to build a memristive architecture of the chunking model and demonstrate the feasibility, which provides neuromorphic engineers with a promising solution to realize dynamical memory on hardware platform.

We fabricate a *FeO*_*x*_-based memristor device with typical sandwich structure, whose detailed process and electrical characteristics are shown in Figure S1. The conductance state can be monotonously and consecutively modulated under a series of positive or negative pulses, i.e., with good plasticity. The positive pulse train gradually increases the conductance, corresponding to the short/long term potentiation (STP/LTP) process; while the negative pulse train results in short/long term depression (STP/LTD). The resistive ratio of highest conductance to lowest conductance is only 3 ~ 4.

The memristor is cascaded with an amplifier to perform as a functional synapse. The one-input-to-one-output structure and multiple-input-to-one-output structure are illustrated in Figures S2A,B, respectively. The equivalent synaptic weight is co-determined by the memristor conductance *G* and feedback resistance *R*_*f*_ on amplifier, *w* = *V*_*out*_/*V*_*in*_ = −*R*_*f*_*G*, which is a dimensionless value indicating the voltage transmission efficiency from input to output. In this manner, the negative weight realizes the inhibitory connection in HCSM model. For the case of multiple inputs injected to one amplifier, all the memristors form a parallel circuit and the transfer function is provided in Figure S2. The amplifier is able to accumulate the multiple synaptic inputs, like the integration function of dendrites. This feature efficiently supports the multiplication and accumulation (MAC) operations between the inputs and weights in Equation (2).

The neuron dynamics described by the differential equation (Equation 2) can be numerically solved based on its corresponding difference equation

(6) $$\begin{array}{l} x_{i}^{\left(n,m\right)}(t+dt)=x_{i}^{\left(n,m\right)}(t)+dt\cdot \{\tau^{\left(n,m\right)}x_{i}^{\left(n,m\right)}(t)\cdot [\sigma_{i}^{\left(n,m\right)}\\ \;-\;\sum_{j\;=\;1}^{N_{0}}w_{ij}^{\left(n,m\right)}\cdot x_{j}^{\left(n,m\right)}(t)]+v_{i}^{\left(n,m\right)}\}. \end{array}$$

If we replace the evolution “*t* → *t* + *dt*” by “*k* → *k* + 1,” we can achieve a numerical iteration process. The one-to-one corresponding digital neuron block is shown in Figure S3A. A Fibonacci sequence block is also necessary to determine $\sigma_{i}^{\left(n,m\right)}$, as well as the upper and lower bounds of the synaptic weights, as shown in Figure S3B.

Based on the element synapse and neuron block, a chunk network can be implemented, as demonstrated in Figure 3. Each chunk, a PC or a CC, is mainly constructed by an analog memristor crossbar circuit and digital neurons. More specifically, the weighted synapses which is the most critical part in this neural network, are implemented by a memristor crossbar circuit. Each column in the memristor crossbar and the cascaded amplifier on that column perform a MAC operations as shown in Figure S2B. All columns are assumed to be independent without crosstalk, so that the whole memristor crossbar and the amplifier array can well realize the matrix-vector multiplication (VMM) which is the major operation in neural networks. This indicates that the architecture supports one-time projection from multiple inputs to multiple outputs, with the advantages of small size, high speed and low power. It is worth noting that the diagonal elements of the crossbar are non-plastic resistors (not memristor) with the same value as the feedback resistance *R*_*f*_ on the amplifier. Thus, the requirement of *w*_*ii*_ = 1 in HCSM model is met. Each neuron block iteratively runs the dynamics of Equation (6). The neuronal outputs at each time step are stored in temporal registers and fed back into the network as synaptic inputs at the next time step. In fact, the timing sequence of the whole network (*k* → *k* + 1…) is governed by the clock signal. Actually, the chunk is an analog-digital hybrid circuits, DACs (digital to analog converters) and ADCs (analog to digital converters) are required to convert the signal format (Li et al., 2013). Furthermore, each chunk circuit can be hierarchically organized together to form a complete HCSM model, as shown in Figure S4. The winner neuron activator in each chunk (PC) is used to determine the winner neuron at each time step and transmit an excitatory signal to the corresponding connected CC circuit. When a CC receives an excitatory activation signal from a winner neuron in the PC, the clock will be triggered and the iterative neuronal dynamics in this CC starts to form a pre-defined memory trace.

When performing a real task, the memristive networks often work in two stages: the write (synaptic modulation) stage and the read (neuronal processing) stage. During the write stage, the memristive crossbar is fully controlled by the pulse modulator block, as presented in Figure S5. The weight calculator block calculates the theoretical weight of each synapse according to a pre-defined chunking sequence based on Equations (3)–(5), and the pulse modulator generates the pulse train (potentiation or depression pulses) to modify the conductance of each memristor to the desired value. Two detailed modulation methods are illustrated in Figures S6, S7. Different from the conventional direct configuration in computer software, neuromorphic implementation has to gradually program the conductance of hardware synapse array from a random initial state to the target state that is produced by the weight calculator. Pulse tuning scheme is more popular, compared to DC switching, since its well controllable modulating increment can achieve relatively high precision. The open-loop modulation directly uses the behavior model of memristor device to determine the direction and number of pulses to move any initial conductance state to the desired one. Considering real device variability, one-time open-loop modulation sometimes cannot reach the ideal state. To this end, the closed-loop modulation repeatedly performs the open-loop modulation until the desired conductance is achieved. More generally, the closed-loop modulation can use the trial-and-error method to gradually tune the conductance without the guidance of theoretical behavior model. Furthermore, the modulation process is flexible by choosing proper pulse amplitude (Kuzum et al., 2011), width (Snider, 2008), and frequency (He et al., 2014) of the pulse train. However, the versatile pulse tuning schemes will drastically increase the burden of the pulse generator, hence it is often hard to be executed in hardware systems. To mitigate the burden, a number of identical pulses are adopted in this work to modulate the memristor states, as mentioned earlier in Figure S1C.

When updating the conductance of a specific memristor in the crossbar, it would be firstly selected to avoid influencing the states of other unselected memristors. Some typical selective devices are useful such as diode or transistor (Wong et al., 2012), and even selector-free memristor crossbar is possible (Prezioso et al., 2015). The half-selected technique is also used to further prevent unintentional operation on the unselected memristors (Yang et al., 2013). This is because the conductance change only occurs when the amplitude of modulation pulses is above the threshold voltage and no significant conductance change is observed under applied voltages below the threshold (Jo et al., 2010). The full programming voltage *V*_*P*_ and *V*_*D*_ on the selected memristor is above the threshold, while the half voltage *V*_*P*_/2 and *V*_*D*_/2 is configured below the threshold. Then while one synapse is under programming, the others are clamped at their current states with a lower half-selected voltage. During the read stage, the pulse modulator is switched off and the data flows from the memristor crossbar to the neuron block, and then feeds back at next time step. The input voltages are scaled to be sufficiently small that the trained memristor states would not change during the whole read stage.

This paper aims for offering a heuristic solution to guide neuromorphic engineers to embed a dynamical memory model into future neuromorphic platforms. In all the following simulations, we present less peripheral circuit details but pay more attention to the influence of dynamic range and precision of memristor device, which are the two key points narrowing the gap between memristive system and HCSM model. Based on the real memristor data in Figure S1, as well as some existing physical models and behavioral models of the memristor (Strukov et al., 2008; Yang et al., 2008; Guan et al., 2012a; Suri et al., 2012; Deng et al., 2015), we build an iron oxide memristor model whose synaptic behavior shows excellent agreement with the real device experiments (Figure S1D). Furthermore, we use SPICE (a standard circuit simulator) to verify the proposed network model of HCSM shown in Figure 3 and Figure S4.

## 3. Results

### 3.1. Chunking and synaptic plasticity

We denote $\text{sup}\left(S_{1}^{\left(n,m\right)}\right)$ as the supremum of set *S*_1_ defined in Equation (5) in the chunk (*n, m*), and $\text{inf}\left(S_{2}^{\left(n\prime ,m\prime \right)}\right)$ as the infimum of set *S*_2_ in the chunk (*n*′, *m*′). The ratio of $\text{inf}\left(S_{2}^{\left(n\prime ,m\prime \right)}\right)$ to $\text{sup}\left(S_{1}^{\left(n,m\right)}\right)$ generally implies the requirement of synaptic plasticity to recall the sequences. As there are multiple chunks in different layers, a relative synaptic plasticity requirement index φ is defined as

(7) $$\begin{array}{l} \varphi =\frac{\max\{\inf(S_{2}^{\left(n\prime ,\;m\prime \right)})\}}{\min\{\sup(S_{1}^{\left(n,\;m\right)})\}}=\frac{g^{\max\{k^{\left(n,\;m\right)}\}-1}+1}{g}=g^{\max\{k^{\left(n,\;m\right)}\}-2}+g^{-1} \end{array}$$

where max{**k**^(*n, m*)^} is the length of the longest chunking sequence for a particular memory task. It is known that φ in real neurobiological systems should be less than an upper bound, which constitutes the capacity boundary of sequential memory. As mentioned previously, the dynamic range of memristor with good synaptic plasticity is often relatively narrow. This may relate to capacity limitations in the human brain. Note that in the HCSM, a sequence is divided into a series of subsequences with different lengths, and the chunk with the longest sequence mainly determines the requirement on the dynamic range of the memristor. In this regard, the HCSM model is capable of having the neuromorphic system maintaining its performance with a reduced requirement of synaptic plasticity.

**Figure 4:**
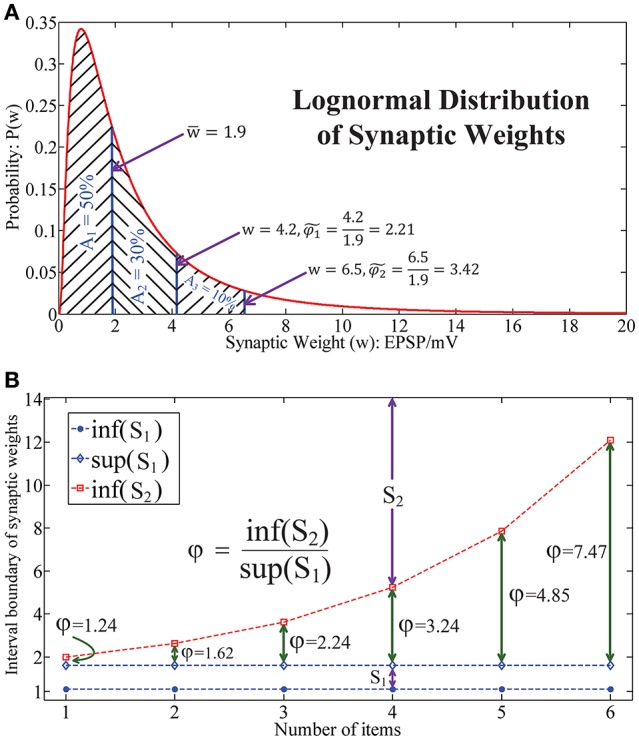
**Lognormal distribution of synaptic weights underlies the chunking mechanism. (A)** The lognormal distribution of synaptic weights in real neural systems, which implies that the dynamic range of the synapses in the brain is in a narrow interval. **(B)** The relative synaptic plasticity requirement index with respect to the length of the sequence in a particular chunk for HCSM.

As shown in the literature, the synaptic weight distribution (Barbour et al., 2007) in the human brain follows a lognormal distribution (Song et al., 2005; Teramae and Fukai, 2014), as illustrated in Figure 4A. This indicates that the synaptic weights mainly locate in a narrow domain. Note that generally it is impossible to estimate the relative synaptic plasticity requirement index φ in human brain and bio-neural systems by applying (Equation 7). Here we define another index $\tilde{\varphi }$ to address this issue. Let $\bar{w}$ be the median weight and define $\tilde{\varphi }=\frac{w}{\bar{w}}$ as the measurement of relative synaptic plasticity index in bio-neural systems. Thus, in Figure 4A, it is observed that for 80 and 90% of synaptic weights, the synaptic plasticity index is below ${\tilde{\varphi }}_{1}=\frac{4.2}{1.9}=2.21$ and ${\tilde{\varphi }}_{2}=\frac{6.5}{1.9}=3.42$, respectively.

While in Figure 4B, it is seen that φ increases exponentially with respect to the length of the sequence in HSCM. As a chunking mechanism allows us to encode a long sequence into many shorter subsequences, HCSM could work on the domain which requires a relative narrower dynamic range of the memristor in the neuromorphic network. By combining the results shown in both Figures 4A,B, when we apply $\tilde{\varphi }$ to reflect the requirement of synaptic plasticity in the brain/bio-neural systems, we provide a putative reason why the optimal items in sequential memory is 3–4 items, which is a long standing problem pointed out in (Simon, 1974) and (Gobet, 2004).

### 3.2. Impact on the precision of the synaptic weights in HCSM

**Figure 5:**
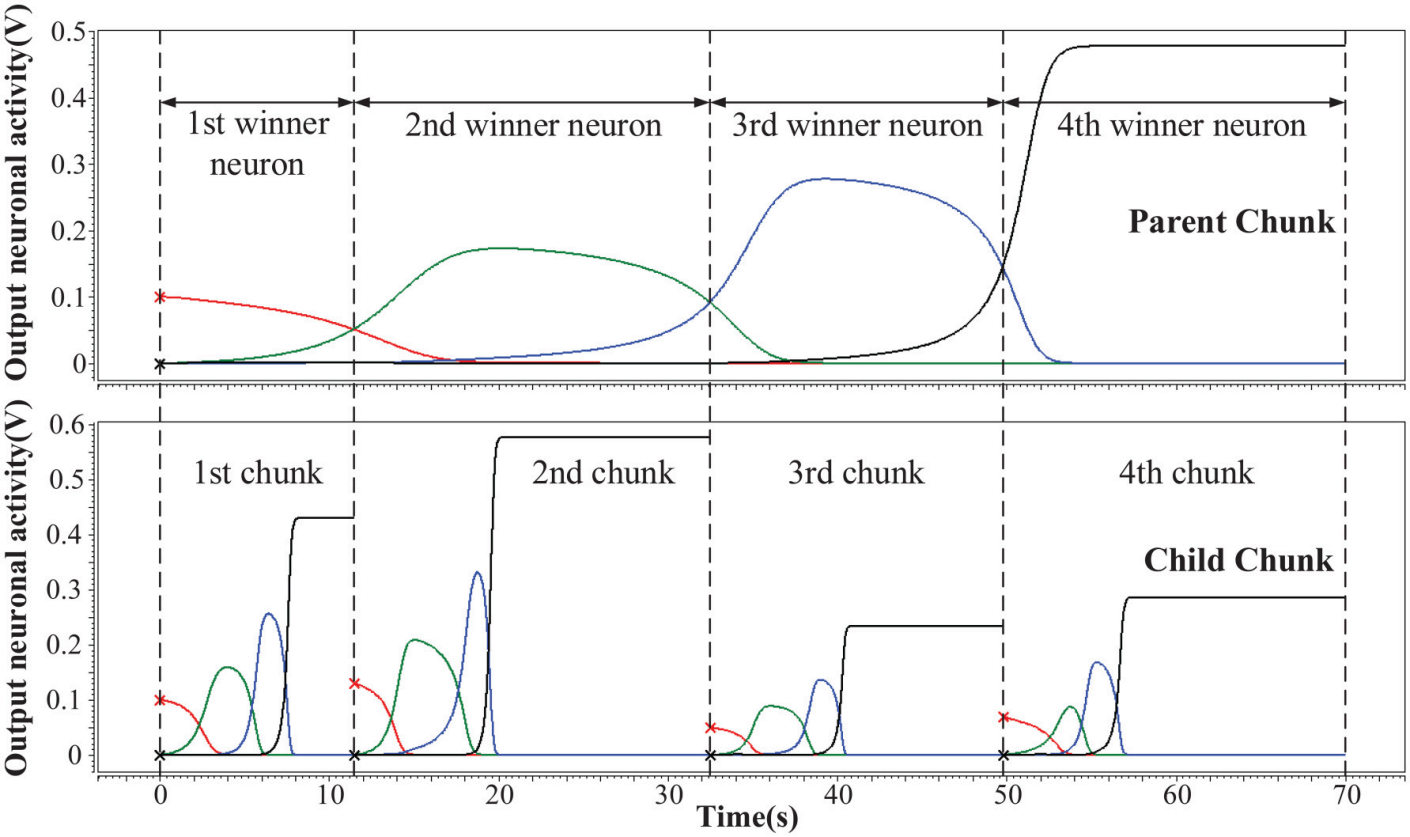
**Hierarchical memory traces in one PC and its CCs**. The vertical axis is the output neuronal activity and the horizontal axis is the sampling time. At a given moment, the neuron which preserves the maximum activity is the temporal winner neuron.

We simulate the hardware implementation of HCSM (Figure 3) and the results of neuronal activities are shown in Figure 5, where an example consisting of four winner neurons in each chunk is illustrated. Specifically, several sub-circuits with the same structure but different parameters, each represents a PC or a CC, have been established to form the complete HCSM model. A 50 Hz square wave is provided as the gated clock signal for all sub-circuits, while each gate is controlled by its activation signal. The clock gate is on when the activation signal is logically high. Then the sub-circuit is activated. The computation module of the activated sub-circuit, consisting of a memristor array, a group of neurons and other peripheral circuits, is then triggered by the 50 Hz clock signal. Output neuronal activities of the activated subcircuit gradually evolve following Equation (6) where each time step is kept smaller than a half clock period. The winner neuron activator then determines the winner neuron with maximum neuronal activity and set the corresponding activation signal logically high to activate its connected CC. It is seen that the ideal memory trace is successfully achieved, where different neurons become the temporal winner in turn. Thus, the trace in the CCs is instantly activated by a corresponding activation signal generated from the PC.

**Figure 6:**
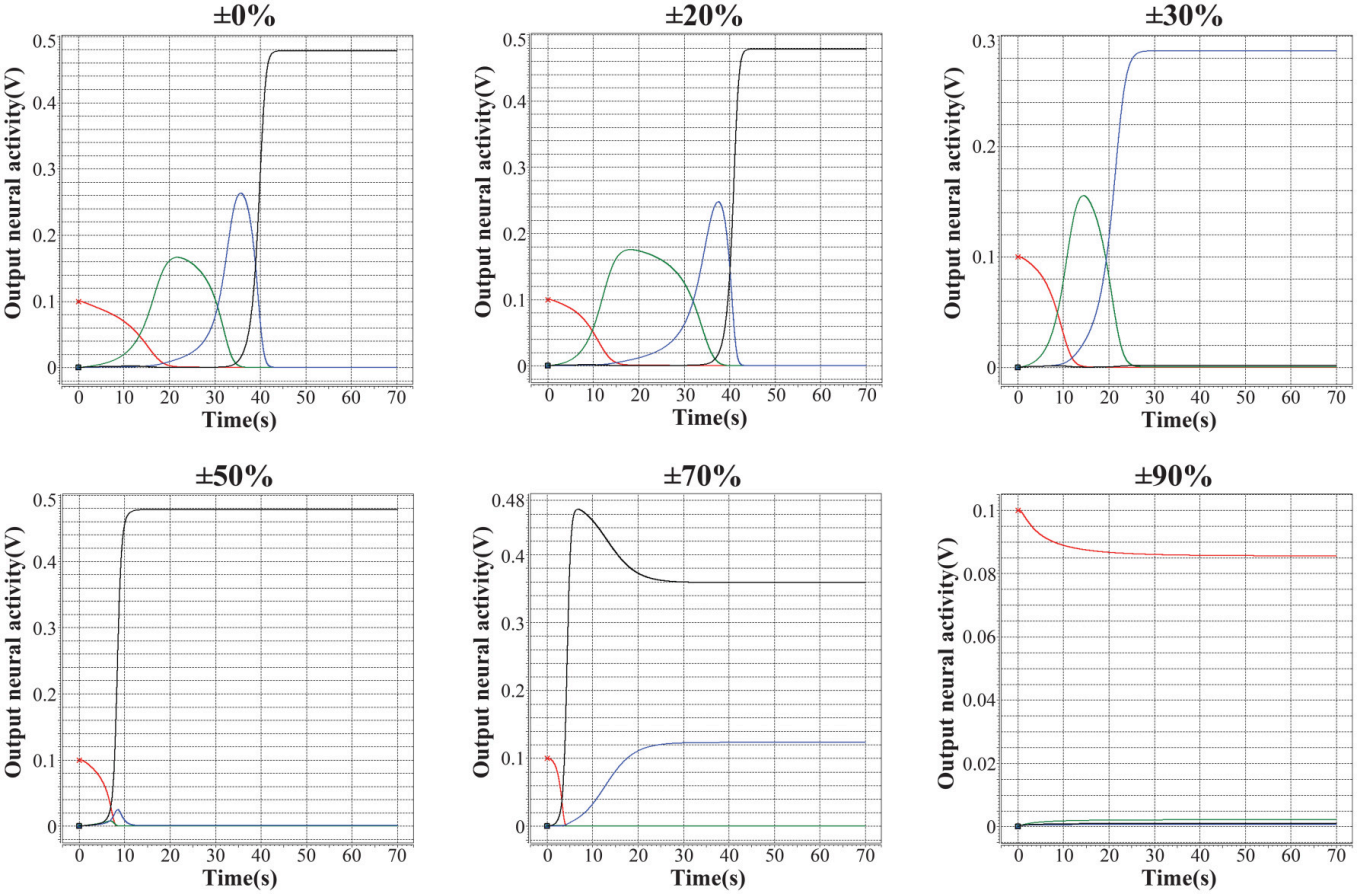
**Analysis of the fault-tolerance performance of the proposed HCSM system with respect to the variation of memristors**. It can be seen that although with a weight dispersion of up to 20%, the HCSM system can perfectly pass by all the four temporal winners. And the system can pass by three of the four temporal winners even with a dispersion of 30%. Hence, a good tolerance of the proposed HCSM system to the device variation can be shown.

As discussed in (Kuzum et al., 2012), (Yu et al., 2013), (Guan et al., 2012b), and (Yu et al., 2012), the main challenge of memristor-based neuromorphic system is the notable variation of memristive devices during programming, including cycle-to-cycle variation and device-to-device variation. In this case, the performance of the encoded memristor-based neuromorphic network of HCSM in the presence of device variation need to be validated. We introduce different levels of fuzzy dispersion to the final weight values of synaptic weights in HCSM using our fabricated memristor as synapse when recalling a memory trace task. Figure 6 shows the robustness of HCSM model by analyzing the fault-tolerance performance with respect to the weight variation of memristors. In particular terms, the network can perfectly trace the target sequence under a pessimistic 20% dispersion of the synaptic weights. As expected, the responses of pre-defined winner neurons gradually deviate from the ideal pattern with a rapid increasement of weight dispersion. For example, only three winner neurons successfully trace its memory under 30% dispersion of the synaptic weights, and the number of successful neurons reduces to two when the dispersion level increases to 50%. The trace pattern no longer converges to its stable state when the dispersion is larger than 70%. In general, our proposed HCSM model does not require precise synaptic weights in the encoding scheme, and a great degree of device variation can be tolerated. This suggests that chunking mechanism enables applying low precision synapses when performing a memory task.

### 3.3. Impact on the dynamic range of the synaptic weight in HCSM

In simulating the encoding process of a sequential memory on SPICE, two conclusions are obtained: (i) the dynamic range of the synaptic weight is required to be much wider if the same length of sequential memory is encoded without chunking; (ii) the success rate of the encoding in each chunk is a monotonously increasing function of the dynamic range synaptic weights.

Suppose that we encode a sequential memory with **k** items such that the square root of **k** is an integer. To achieve a lower relative synaptic plasticity requirement index defined in Equation (7), the best way is to encode the sequence in $\sqrt{\text{k}}$ chunks, with each chunk consisting $\sqrt{\text{k}}$ items. Then, the relative synaptic plasticity requirement index φ is obtained by

(8) $$\begin{array}{l} \varphi =\frac{\max\{\inf(S_{2}^{\left(n\prime ,\;m\prime \right)})\}}{\min\{\sup(S_{1}^{\left(n,\;m\right)})\}}=g^{\sqrt{k}-2}+g^{-1} \end{array}$$

By comparing Equations (7) and (8), it is seen that we require the same dynamic range of the synaptic weight to encode **k** items with chunking mechanism and $\sqrt{\text{k}}$ items without chunking mechanism.

**Figure 7:**
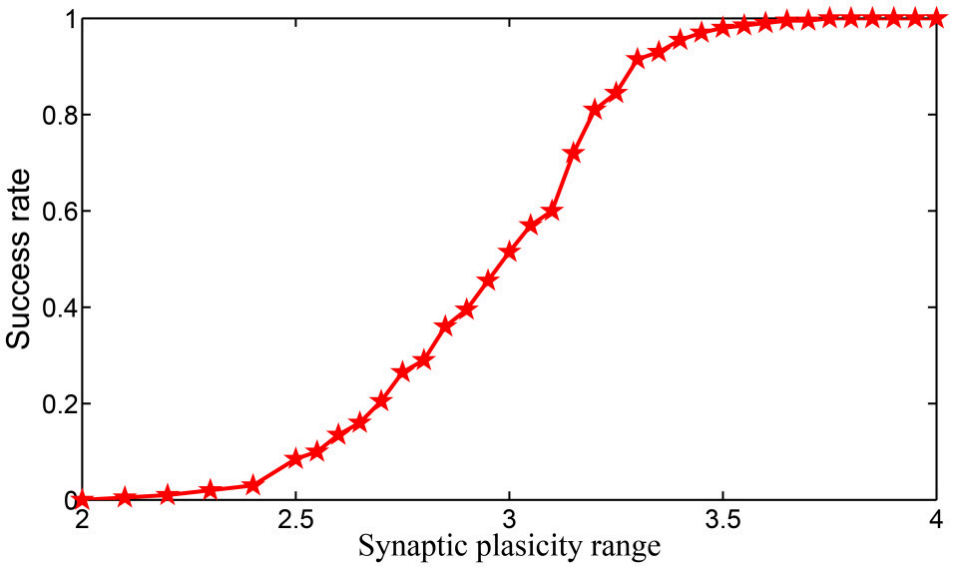
**The relationship of encoding success rate and the dynamic range of synaptic weights**.

We simulate the encoding process of a length of 16-items sequential memory which has 4 chunks, with each chunk consisting of 4 items on SPICE based on the encoding scheme we introduced in Equations (3)–(5). In Equation (5), we notice that the supremum of set *S*_2_ can be positive infinity. However, in real applications it is well known that the synaptic weight can never be infinity. To show the impact of the dynamic range of the synaptic weight in HCSM more clearly, we set |*S*_2_| = |*S*_1_| where |.| denotes the measure/length of an interval, i.e., $S_{2}=\left\{x|g^{\text{k}\;-\;1}+1<x<g^{\text{k}\;-\;1}+\frac{3}{2}\right\}$. Obviously, we have $|S_{2}|=|S_{1}|=\frac{1}{2}$. When both |*S*_1_| and |*S*_1_| are fixed, the relative synaptic plasticity requirement index φ also reflects the requirement of the dynamic range of synaptic weights. In SPICE simulation, φ is chosen from 2–4. In Figures S8–S11 in Supplementary Information, the effects for cases φ = 2.0, 2.4, 3, and 3.6 are shown in four figures, respectively. It is seen that a small φ usually leads to failure of the encoding of the sequential memory while a larger φ improves such a situation. We repeated the experiments 200 times to estimate the encoding success rate for each fixed φ in Figure 7, where it is shown that the encoding success rate in each chunk is a monotonously increasing function of the dynamic range of synaptic weights.

## 4. Discussion

In this work, we suggest a link between chunking mechanism and synaptic plasticity to answer a long standing question why chunking improves sequential memory. A hierarchical chunking of sequential memory (HCSM) model and a robust scheme regarding how to encode sequential memory are presented. It is observed from the encoding scheme that chunking mechanism reduces the requirements of synaptic plasticity when recalling a memory trace, including the tolerance of the dynamic range and precision of the synaptic weights. Furthermore, we provide a neuromorphic implementation to verify the proposed memory dynamics under the hardware constraints of narrow dynamic range and device variability. The successful demonstration indicates the feasibility to embed more complex memory models into future neuromorphic systems.

One merit of the proposed HCSM model is the robustness of the encoding method, i.e., the weight can be a random value in a given interval, which makes the model convenient to be realized by memristive devices. However, the disadvantages include (i) the model requires full connection of the neurons in the network; and (ii) the asymmetry in information storage fundamentally impairs the length of the memory trace. Therefore, the investigation of a new model that allows sparse connection of neurons to link metastable states together in a sequential memory would be of great interest. Also, besides chunking mechanism, how can the memory capacity of biological systems be improved deserves investigation. Furthermore, we would like to point out that in HCSM, a particular item in the memory trace is represented by only one neuron. However, experimental studies have revealed that population coding (Pasupathy and Connor, 2002), a method to represent stimuli (a memory item) by using the joint activities of a number of neurons, is widely used in the brain (Averbeck et al., 2006). This implies that single neural coding method may be inadequate in practical applications. We conjecture that population coding could be applied to our model which deserves further investigations.

This work provides an addition to recent work on learning of chunking sequences (Fonollosa et al., 2015) including specific roles in cognitive process (Varona and Rabinovich, 2016). Specifically, this work provides a useful hardware validation means for many advanced theory researches. We also open up a new application space on neuromorphic platforms to implement not only HCSM, but also various bio-inspired memory models related to the encoding of the visual, acoustic and semantic information and so on. Predictably, the disciplines of cognitive psychology, neuroscience and information technology, and neuromorphic engineering becomes more and more important. The top-down bio-plausible theories fundamentally guide the development of future neuromorphic computing systems; while the bottom-up neuromorphic materials, chips, boards and systems usefully verify these pioneering theories. Although there is still a long road ahead, this work kindles a ray of hope.

The major difficulty preventing its application is the fabrication and management of large-scale memristor crossbar, especially considering the device variability and the crosstalk among adjacent cells. On the other side, the required peripheral circuits are also quite complex, including analog-digital converters, read/write circuits, switching matrix as well as extra computing circuits for learning. Fortunately, some reported memristor-based artificial neural networks have shown that these developments may become feasible in the near future, at least in relatively small scale (Alibart and Zamanidoost, 2013; Garbin et al., 2014; Prezioso et al., 2015). With the development of integration techniques for large scale memristor crossbar or even 3D networks (Yu et al., 2013; Li et al., 2016), as well as memristor for logical or arithmetical computations (Borghetti et al., 2010; Gale, 2015) to reduce complex peripheral circuits by replacing the digital neurons, we envisage a real chip able to perform interesting memory tasks.

## Author contributions

GL, LD, and SS proposed the model and designed the experiments. DW and WW did the SPICE simulation. ZZ, HL, and FZ did the device experiments. JP and LS contributed to the main idea of this paper. All authors write the manuscript.

### Conflict of interest statement

The authors declare that the research was conducted in the absence of any commercial or financial relationships that could be construed as a potential conflict of interest.

## References

[B1] Alibart F. Zamanidoost E. Strukov, D. B. (2013). Pattern classification by memristive crossbar circuits using *ex situ* and *in situ* training. Nat. Commun. 4:2072 10.1038/ncomms3072 23797631

[B2] Alibart F. Pleutin S. Bichler O. Gamrat C. Serrano-Gotarredona T. Linares-Barranco B. (2012). A memristive nanoparticle/organic hybrid synapstor for neuroinspired computing. Adv. Funct. Mater. 22, 609–616. 10.1002/adfm.201101935

[B3] Averbeck B. B. Latham P. E. Pouget A. (2006). Neural correlations, population coding and computation. Nat. Rev. Neurosci. 7, 358–366. 10.1038/nrn1888 16760916

[B4] Barbour B. Brunel N. Hakim V. Nadal J. P. (2007). What can we learn from synaptic weight distributions? Trends Neurosci. 30, 622–629. 10.1016/j.tins.2007.09.005 17983670

[B5] Bernacchia A. Seo H. Lee D. Wang X. J. (2011). A reservoir of time constants for memory traces in cortical neurons. Nat. Neurosci. 14, 366–372. 10.1038/nn.2752 21317906 PMC3079398

[B6] Bi G. Q. Poo M. M. (1998). Synaptic modifications in cultured hippocampal neurons: dependence on spike timing, synaptic strength, and postsynaptic cell type. J. Neurosci. 18, 10464–10472. 9852584 10.1523/JNEUROSCI.18-24-10464.1998 PMC6793365

[B7] Bick C. Rabinovich, M. I. (2009). Dynamical origin of the effective storage capacity in the brains working memory. Phys. Rev. Lett. 103, 218101–218104. 10.1103/PhysRevLett.103.218101 20366069

[B8] Borghetti J. Snider G. S. Kuekes P. J. Yang J. J. Stewart D. R. Williams R. S. (2010). ‘Memristive’ switches enable ‘stateful’ logic operations via material implication. Nature 464, 873–876. 10.1038/nature08940 20376145

[B9] Brunel N. Wang X. J. (2001). Effects of neuromodulation in a cortical network model of object working memory dominated by recurrent inhibition. J. Comput. Neurosci. 11, 63–85. 10.1023/A:1011204814320 11524578

[B10] Carrillo-Reid L. Lopez-Huerta V. G. Garcia-Munoz M. Theiss S. Arbuthnott G. W. (2015). Cell assembly signatures defined by short-term synaptic plasticity in cortical networks. Int. J. Neural. Syst. 25:1550026. 10.1142/S0129065715500264 26173906

[B11] Chang T. Jo S. H. Lu W. (2011). Short-term memory to long-term memory transition in a nanoscale memristor. ACS Nano 5, 7669–7676. 10.1021/nn202983n 21861506

[B12] Chen Z. Cowan N. (2005). Chunk limits and length limits in immediate recall: a reconciliation. J. Exp. Psychol. Learn. Mem. Cogn. 31, 1235–1249. 10.1037/0278-7393.31.6.1235 16393043 PMC2673719

[B13] Chua L. O. (1971). Memristor-the missing circuit element. IEEE Trans. Circuit Theory 18, 507–519. 10.1109/TCT.1971.1083337

[B14] Chua L. O. (2011). Resistance switching memories are memristors. Appl. Phys. A 102, 765–783. 10.1007/s00339-011-6264-9

[B15] Cowan N. (2001). The magical number 4 in short-term memory: a reconsideration of mental storage capacity. Behav. Brain Sci. 24, 87–114. 10.1017/S0140525X01003922 11515286

[B16] Deng L. Li G. Deng N. Wang D. Zhang Z. He W.. (2015). Complex learning in bio-plausible memristive networks. Sci. Rep. 5:10684. 10.1038/srep10684 26090862 PMC4473596

[B17] Deng L. Wang D. Zhang Z. Tang P. Li G. Pei J. (2016). Energy consumption analysis for various memristive networks under different learning strategies. Phys. Lett. A 380, 903–909. 10.1016/j.physleta.2015.12.024

[B18] Dunlap R. A. (1997). The Golden Ratio and Fibonacci Numbers. World Scientific Publishing.

[B19] Edin F. Klingberg T. Johansson P. McNab F. Tegnér J. Compte A. (2009). Mechanism for top-down control of working memory capacity. Proc. Natl. Acad. Sci. U.S.A. 106, 6802–6807. 10.1073/pnas.0901894106 19339493 PMC2672558

[B20] Fonollosa J. Neftci E. Rabinovich M. (2015). Learning of chunking sequences in cognition and behavior. PLoS Comput. Biol. 11:e1004592. 10.1371/journal.pcbi.1004592 26584306 PMC4652905

[B21] Gale E. (2015). Single memristor logic gates: from NOT to a full adder. *arXiv*: 1510.05705 27199243

[B22] Garbin D. Bichler O. Vianello E. Rafhay Q. Gamrat C. Perniola L. (2014). Variability-tolerant convolutional neural network for pattern recognition applications based on oxram synapses, in IEEE International Electron Devices Meeting (IEDM) (San Francisco, CA), 28.4.1–28.4.4. 10.1109/IEDM.2014.7047126

[B23] Gholipour A. Kehtarnavaz N. Briggs R. Devous M. Gopinath K. (2007). Brain functional localization: a survey of image registration techniques. IEEE Trans. Med. Imaging 26, 427–451. 10.1109/TMI.2007.892508 17427731

[B24] Gobet F. Lane P. C. Croker S. Cheng P. Jones G. Oliver I.. (2001). Chunking mechanisms in human learning. Trends Cogn. Sci. 5, 236–243. 10.1016/S1364-6613(00)01662-4 11390294

[B25] Gobet F. Clarkson G. (2004). Chunks in expert memory: evidence for the magical number four or is it two? Memory 12, 732–747. 10.1080/09658210344000530 15724362

[B26] Guan X. Yu S. Wong H. S. P. (2012a). A SPICE compact model of metal oxide resistive switching memory with variations. IEEE Electr. Device Lett. 33, 1405–1407. 10.1109/LED.2012.2210856

[B27] Guan X. Yu S. Wong H. S. P. (2012b). On the switching parameter variation of metal-oxide RRAM-part I: physical modeling and simulation methodology. IEEE Trans. Electron. Dev. 59, 1172–1182. 10.1109/TED.2012.2184545

[B28] Han F. Wiercigroch M. Fang J. A. Wang Z. (2011). Excitement and synchronization of small-world neuronal networks with short-term synaptic plasticity. Int. J. Neural. Syst. 21, 415–425. 10.1142/S0129065711002924 21956933

[B29] He W. Huang K. Ning N. Ramanathan K. Li G. Jiang Y.. (2014). Enabling an integrated rate-temporal learning scheme on memristor. Sci. Rep. 4:4755. 10.1038/srep04755 24755608 PMC3996481

[B30] Hebb D. O. (1949). The Organization of Behavior: A Neuropsychological Theory. New York, NY: John Wiley and Sons.

[B31] Huntley J. Bor D. Hampshire A. Owen A. Howard R. (2011). Working memory task performance and chunking in early Alzheimers disease. Brit. J. Psychiat. 198, 398–403. 10.1192/bjp.bp.110.083857 21525522

[B32] Jackson B. L. Rajendran B. Corrado G. S. Breitwisch M. Burr G. W. Cheek R. (2013). Nanoscale electronic synapses using phase change devices. ACM J. Emerg. Tech. Com. 9:12 10.1145/2463585.2463588

[B33] Jo S. H. Chang T. Ebong I. Bhadviya B. B. Mazumder P. Lu W. (2010). Nanoscale memristor device as synapse in neuromorphic systems. Nano Lett. 10, 1297–1301. 10.1021/nl904092h 20192230

[B34] Kim H. Sah M. P. Yang C. Roska T. Chua L. O. (2012). Memristor bridge synapses. Proc. IEEE 100, 2061–2070. 10.1109/JPROC.2011.2166749

[B35] Kreiman G. Fried I. Koch C. (2001). Single neuron responses in humans during binocular rivalry and flash suppression. J. Vision 1:131 10.1167/1.3.131

[B36] Kreiman G. Koch C. Fried I. (2000). Category-specific visual responses of single neurons in the human medial temporal lobe. Nat. Neurosci. 3, 946–953. 10.1038/78868 10966627

[B37] Kuzum D. Jeyasingh R. G. Lee B. Wong H. S. (2011). Nanoelectronic programmable synapses based on phase change materials for brain-inspired computing. Nano Lett. 12, 2179–2186. 10.1021/nl201040y 21668029

[B38] Kuzum D. Jeyasingh R. G. D. Yu S. Wong H. S. (2012). Low-energy robust neuromorphic computation using synaptic devices. IEEE Trans. Electron. Dev. 59, 3489–3494. 10.1109/TED.2012.2217146

[B39] Kuzum D. Yu S. Wong H. S. P. (2013). Synaptic electronics: materials, devices and applications. Nanotechnology 24:382001. 10.1088/0957-4484/24/38/382001 23999572

[B40] Li H. Li K. S. Lin C. H. Hsu J. L. Chiu W. C. Chen M. C. (2016). Four-layer 3D vertical RRAM integrated with FinFET as a versatile computing unit for brain-inspired cognitive information processing, in IEEE Symposium on VLSI Technology (Honolulu, HI). 10.1109/vlsit.2016.7573431

[B41] Li B. Shan Y. Hu M. Wang Y. Chen Y. Yang H. (2013). Memristor-based approximated computation, in Proceedings of the 2013 International Symposium on Low Power Electronics and Design (ISLPED) (Beijing: IEEE Press), 242–247. 10.1109/islped.2013.6629302

[B42] Lindley R. H. (1966). Recoding as a function of chunking and meaningfulness. Psychon. Sci. 6, 393–394. 10.3758/BF03330953

[B43] Livio M. (2008). The Golden Ratio: the Story of Phi, the World's Most Astonishing Number. Random House LLC.

[B44] Maass W. (2014). Noise as a resource for computation and learning in networks of spiking neurons. Proc. IEEE 102, 860–880. 10.1109/JPROC.2014.2310593

[B45] Mante V. Sussillo D. Shenoy K. V. Newsome W. T. (2013). Context-dependent computation by recurrent dynamics in prefrontal cortex. Nature 503, 78–84. 10.1038/nature12742 24201281 PMC4121670

[B46] Mead C. (1989). Analog VLSI Implementation of Neural Systems. Portland: Addison-Wesley.

[B47] Miller G. A. (1956). The magical number seven, plus or minus two: some limits on our capacity for processing information. Psychol. Rev. 63, 81–97. 10.1037/h0043158 13310704

[B48] Pasupathy A. Connor C. E. (2002). Population coding of shape in area V4. Nat. Neurosci. 5, 1332–1338. 10.1038/972 12426571

[B49] Prezioso M. Merrikh-Bayat F. Hoskins B. D. Adam G. C. Likharev K. K. Strukov D. B. (2015). Training and operation of an integrated neuromorphic network based on metal-oxide memristors. Nature 521, 61–64. 10.1038/nature14441 25951284

[B50] Rabinovich M. I. Huerta R. Laurent G. (2008). Transient dynamics for neural processing. Science 321, 48–50. 10.1126/science.1155564 18599763

[B51] Rabinovich M. I. Huerta R. Varona P. Afraimovich V. S. (2008). Transient cognitive dynamics, metastability, and decision making. PLoS Comput. Biol. 4:e1000072. 10.1371/journal.pcbi.1000072 18452000 PMC2358972

[B52] Rabinovich M. I. Varona P. Tristan I. Afraimovich V. S. (2014). Chunking dynamics: heteroclinics in mind. Front. Comput. Neurosci. 8:22. 10.3389/fncom.2014.00022 24672469 PMC3954027

[B53] Rabinovich M. Volkovskii A. Lecanda P. Huerta R. Abarbanel H. D. I. Laurent G. (2001). Dynamical encoding by networks of competing neuron groups: winnerless competition. Phys. Rev. Lett. 87, 0681021–0681024. 10.1103/PhysRevLett.87.068102 11497865

[B54] Ramanathan K. Ning N. Dhanasekar D. Li G. Shi L. Vadakkepat P. (2012). Presynaptic learning and memory with a persistent firing neuron and a habituating synapse: a model of short term persistent habituation. Int. J. Neural Syst. 22, 12500151–125001520. 10.1142/S0129065712500153 22830965

[B55] Shouval H. Z. Castellani G. C. Blais B. S. Yeung L. C. Cooper L. N. (2002). Converging evidence for a simplified biophysical model of synaptic plasticity. Biol. Cybern. 87, 383–391. 10.1007/s00422-002-0362-x 12461628

[B56] Simon H. A. (1974). How big is a chunk. Science 183, 482–488. 10.1126/science.183.4124.482 17773029

[B57] Snider G. S. (2008). Spike-timing-dependent learning in memristive nanodevices. IEEE Int. Symposium Nanoscale Architect. 2008, 85–92. 10.1109/nanoarch.2008.4585796

[B58] Song S. Miller K. D. Abbott L. F. (2000). Competitive Hebbian learning through spike-timing-dependent synaptic plasticity. Nat. Neurosci. 3, 919–926. 10.1038/78829 10966623

[B59] Song S. Sjöström P. J. Reigl M. Nelson S. Chklovskii D. B. (2005). Highly nonrandom features of synaptic connectivity in local cortical circuits. PLoS Biol. 3:e68. 10.1371/journal.pbio.0030068 15737062 PMC1054880

[B60] Strukov D. B. Snider G. S. Stewart D. R. Williams R. S. (2008). The missing memristor found. Nature 453, 80–83. 10.1038/nature06932 18451858

[B61] Suri M. Bichler O. Querlioz D. Traor B. Cueto O. Perniola L. (2012). Physical aspects of low power synapses based on phase change memory devices. J. Appl. Phys. 112:054904 10.1063/1.4749411

[B62] Tan A. H. Soon H. S. (1996). Concept hierarchy memory model: a neural architecture for conceptual knowledge representation, learning, and commonsense reasoning. Int. J. Neural Syst. 7, 305–319. 10.1142/S0129065796000270 8891845

[B63] Teramae J. Fukai T. (2014). Computational implications of lognormally distributed synaptic weights. Proc. IEEE 102, 500–512. 10.1109/JPROC.2014.2306254

[B64] Tognoli E. Kelso J. A. (2014). The metastable brain. Neuron 81, 35–48. 10.1016/j.neuron.2013.12.022 24411730 PMC3997258

[B65] Varona P. Rabinovich M. I. (2016). Hierarchical dynamics of informational patterns and decision-making. Proc. R. Soc. B 283:20160475. 10.1098/rspb.2016.0475 27252020 PMC4920316

[B66] Wong H. S. P. Lee H. Y. Yu S. Chen Y. S. Wu Y. Chen P. S. (2012). Metal-oxide RRAM. Proc. IEEE 100, 1951–1970. 10.1109/JPROC.2012.2190369

[B67] Yang J. J. Pickett M. D. Li X. Ohlberg D. A. Stewart D. R. Williams R. S. (2008). Memristive switching mechanism for metal/oxide/metal nanodevices. Nat. Nanotechnol. 3, 429–433. 10.1038/nnano.2008.160 18654568

[B68] Yang J. J. Strukov D. B. Stewart D. R. (2013). Memristive devices for computing. Nat. Nanotechnol. 8, 13–24. 10.1038/nnano.2012.240 23269430

[B69] Yu S. Chen H. Y. Gao B. Kang J. Wong H. S. P. (2013). HfOx-based vertical resistive switching random access memory suitable for bit-cost-effective three-dimensional cross-point architecture. ACS Nano 7, 2320–2325. 10.1021/nn305510u 23411406

[B70] Yu S. Gao B. Fang Z. Yu H. Kang J. Wong H. S. P. (2013). A low energy oxide-based electronic synaptic device for neuromorphic visual systems with tolerance to device variation. Adv. Mater. 25, 1774–1779. 10.1002/adma.201203680 23355110

[B71] Yu S. Guan X. Wong H. S. P. (2012). On the switching parameter variation of metal oxide RRAM-part II: model corroboration and device design strategy. IEEE Trans. Electron. Dev. 59, 1183–1188. 10.1109/TED.2012.2184544

[B72] Yu S. Wu Y. Jeyasingh R. Kuzum D. Wong H. S. P. (2011). An electronic synapse device based on metal oxide resistive switching memory for neuromorphic computation. IEEE Trans. Elec. Dev. 58, 2729–2737. 10.1109/TED.2011.2147791

[B73] Zeng H. Chattarji S. Barbarosie M. Rondi-Reig L. Philpot B. D. Miyakawa T.. (2001). Forebrain-specific calcineurin knockout selectively impairs bidirectional synaptic plasticity and working/episodic-like memory. Cell 107, 617–629. 10.1016/S0092-8674(01)00585-2 11733061

