# Supplementary material for: Hierarchical Chunking of Sequential Memory on Neuromorphic Architecture with Reduced Synaptic Plasticity
Source: Front Comput Neurosci. 2016 Dec 20;10:136. doi: 10.3389/fncom.2016.00136 (PMC5168929; doi:10.3389/fncom.2016.00136)
Supplement: Supplementary file 1 [file Presentation1.pdf]

Hierarchical Chunking of Sequential Memory on Neuromorphic Architecture with Reduced Synaptic Plasticity

Supplementary Information

Guoqi Li[*1†], Lei Deng[*1], Dong Wang[*1], Wei Wang[2], Fei Zeng[3], Ziyang Zhang[1], Huanglong Li[1], Sen Song [4], Jing Pei[1] and Luping Shi[1†]

[1] Center for Brain Inspired Computing Research, Department of Precision Instrument, Tsinghua University, Beijing, P.R.China, 100084.

[2] School of Automation Science and Electric Engineering, Beihang University, Beijing, P.R.China, 100191.

[3] Department of materials science and engineering, Tsinghua University, Beijing, P.R.China, 100084.

[4] School of Medicine, Tsinghua University, Beijing, P.R.China, 100084.

[*] The authors contribute equally to this work.

[†] Corresponding: lpshi@mail.tsinghua.edu.cn or liguoqi@mail.tsinghua.edu.cn or peij@mail.tsinghua.edu.cn

1 Theoretical analysis of the existence of metastable states in the memory sequence

Here we assume that there are N_0 neurons in one chunk. Let $I = \{I_1, I_2, ..., I_k, ..., I_{\mathbf{k}_0}\}$ be the set of temporal winners, which forms the memory sequence $\{I_1 \to I_2 \to ... \to I_k \to ... \to I_{\mathbf{k}_0}\}$, and $J = \{I_{\mathbf{k}_0+1}, ..., I_{N_0}\}$ be the set of the remaining neurons. We shall encode $\mathbf{k}_0$ metastable states (i.e., $I = \{I_1, I_2, ..., I_k, ..., I_{\mathbf{k}_0}\}$) as described by the dynamic system in Eq. (2) in the main paper, where I_k denotes the k-th metastable states in the memory sequence. The following Theorem proves the existence of metastable states in the memory sequence using the encoding scheme in Eq. (3)-(5).

Theorem 1. Under the initial activation state $\mathrm{x} = A_k = [0, ..., \sigma_{I_k}, ..., 0] \in R^{N_0 \times 1}$ where σ_{I_k} is the kth entry of A_k, the encoding scheme in Eq. (3)-(5) guarantees the existence of the memory trace of temporal winner neurons $\{I_k \to I_{k+1} \to ... \to I_{\mathbf{k}_0}\}$ in a particular chunk described by the dynamic system in Eq. (2) in the presence of a stimulus with sufficiently small noise.

Proof. The proof is based on the discussion about dissipative saddle point [1]-[3] A of $\dot{\mathrm{x}} = \frac{dE(\mathrm{x})}{d\mathrm{x}}$ with $E(\mathrm{x})$ being the energy function. By omitting (n, m) in the HCSM model, we have

$$E_i(\mathrm{x}) = \frac{1}{2}x_i^2\sigma_i - \frac{1}{2}x_i^2\sum_{j=1,j\neq i}^{N_0} w_{ij}x_j - \frac{2}{3}x_i^3 \quad \text{(S1)}$$

for $i = 1, ...N_0$ and $j = 1, ..., N_0$. Let $\mathrm{Re}(\lambda_1) \geq ... \geq \mathrm{Re}(\lambda_{r-1}) > 0 > \mathrm{Re}(\lambda_r) \geq ... \geq \mathrm{Re}(\lambda_{N_0})$ be the ordered real parts of the eigenvalues of the Hessian matrix $\nabla^2 E(\mathrm{x})$ at A. If the saddle value of A defined as $\nu(A) = \frac{|Re(\lambda_r)|}{Re(\lambda_1)}$ satisfying that

$$\nu(A) > 1 \tag{S2}$$

the saddle point A is dissipative, which implies that there is a contraction of a deviation after the system state passing the neighborhood of the point A . Here we are at the point to prove that the neurons I_k with coordinates A_k for $1 \leq k < \mathbf{k}$ are dissipative while $A_{\mathbf{k}}$ is a stable equilibrium point of the dynamic system in Eq. (2).

The encoding process is to design the σ_i and w_{ij} for $1 \leq i, j \leq N_0$. It can be checked that $A_k = [0, ..., \sigma_{I_k}, ..., 0]$ is a nontrivial fixed point of Eq. (2) and $\nabla^2 E(\mathrm{x})$, which is given by

$$\begin{aligned} [\nabla^2 E(\mathrm{x})]_{ii} &= \sigma_i - 2x_i - \textstyle\sum_{j \neq i} w_{ij} x_j \\ [\nabla^2 E(\mathrm{x})]_{ij} &= -w_{ij} x_i, \ (i \neq j) \end{aligned} \tag{S3}$$

In the beginning, $\mathrm{x} = A_k$ and obviously the neuron I_k is the temporary winner. The eigenvalues of $\nabla^2 E(\mathrm{x})$ at A_k are given as follows:

$$\lambda_i = \begin{cases} \sigma_i - w_{iI_k} \sigma_{I_k}, & \text{if } \ i \in I, i \neq I_{k-1}, I_k, I_{k+1} \\ -\sigma_i, & \text{if } \ i = I_k \\ \sigma_i - w_{iI_k} \sigma_{I_k}, & \text{if } \ i = I_{k-1} \\ \sigma_i - w_{iI_k} \sigma_{I_k}, & \text{if } \ i = I_{k+1} \\ -w_{iI_k} \sigma_{I_k}, & \text{if } \ i \in J \end{cases} \tag{S4}$$

The eigenvalues will be illustrated case by case based on the above equation.

Case 1, $i \in J$:

$$\lambda_i = -w_{iI_k} \sigma_{I_k} < 0 \tag{S5}$$

Case 2, $i \in I$, $i \neq I_{k-1}, I_k, I_{k+1}$:

$$\lambda_i = \sigma_i + w_{iI_k} \sigma_{I_k} < -\sigma_{I_k} < 0 \tag{S6}$$

Case 3, $i = I_{k-1}$:

$$\lambda_i = -\sigma_{I_k} < \sigma_{I_{k-1}} + w_{I_{k-1}I_k}\sigma_{I_k} < 0 \quad \text{(S7)}$$

Case 4, $i = I_{k+1}$:

$$\lambda_i = \sigma_{I_{k+1}} + w_{I_{k+1}I_k}\sigma_{I_k} > 0 \quad \text{(S8)}$$

If $I_k = I_1$, as $\sigma_{I_1} = 1$ and $\sigma_{I_2} = g$, we have

$$\lambda_i = \begin{cases} -1 & \text{if } \ i = 1 \\ \sigma_{I_2} - w_{I_2 I_1}\sigma_{I_1} = g - w_{I_2 I_1}\sigma_{I_1} > 0 & \text{if } \ i = 2 \\ \sigma_i - w_{iI_k}\sigma_{I_k} < 0 & \text{other wise} \end{cases} \quad \text{(S9)}$$

since $w_{iI_1} \in S_1$ if $i = I_2$, otherwise, $w_{iI_1} \in S_2$, where S_1 and S_2 are defined in Eq. (4)-(5).

If $I_k > I_1$, because both $w_{I_{k-1}I_k}$ and $w_{I_{k+1}I_k}$ belongs to S_1, Eq. (3)-(5) implies that

$$(w_{I_{k-1}I_k} + w_{I_{k+1}I_k})\sigma_{I_k} >= 2(g - \tfrac{1}{2})\sigma_{I_k} = 2(\tfrac{\sigma_{I_{k+1}}}{\sigma_{I_k}} - \tfrac{1}{2})\sigma_{I_k} \quad \text{(S10)}$$

as $\frac{\sigma_{I_{k+1}}}{\sigma_{I_k}} = g$. For the Fibonacci sequence, we have

$$2(\tfrac{\sigma_{I_{k+1}}}{\sigma_{I_k}} - \tfrac{1}{2})\sigma_{I_k} = \sigma_{I_{k+1}} + \sigma_{I_{k-1}} \quad \text{(S11)}$$

Then,

$$-\sigma_{I_{k-1}} + w_{I_{k-1}I_k}\sigma_{I_k} > \sigma_{I_{k+1}} - w_{I_{k+1}I_k}\sigma_{I_k} \quad \text{(S12)}$$

i.e.,

$$|\sigma_{I_{k-1}} - w_{I_{k-1}I_k}\sigma_{I_k}| > \sigma_{I_{k+1}} - w_{I_{k+1}I_k}\sigma_{I_k} \quad \text{(S13)}$$

Combining the above four cases and Eq. (S5)-(S13), when either $I_k = I_1$ or $I_k > I_1$ with $k \neq \mathbf{k}_0$, I_k is dissipative and I_{k+1} will be the next temporal winner since only the eigenvalue $\sigma_{I_{k+1}} - w_{I_{k+1}I_k}\sigma_{I_k}$ is positive and its eigenvector pointed to $A_{k+1} = [0, ..., \sigma_{I_{k+1}}, ..., 0]$. Then, the states will go to the

coordinates of the next neuron in the trace

$$\{I_k \rightarrow I_{k+1} \rightarrow ... \rightarrow I_{\mathbf{k}}\} \tag{S14}$$

until it reaches the last one. Note that, though the noise is small, it is necessary to avoid the dynamical system states stopping at a saddle point. For the last temporal winner neuron $I_{\mathbf{k}}$, all eigenvalues of $\nabla^2 E(\mathrm{x})$ at $A_{\mathbf{k}} = [0, ..., \sigma_{I_{\mathbf{k}}}, ..., 0]$ are negative. This implies that $A_{\mathbf{k}}$ is a stable equilibrium point of the dynamic system in Eq. (2).

2 Figures and explanations

Here eight figures are shown in this supplementary information. Figure S1 shows a typical memristor of sandwich structure, and Figure S2 shows the memristor-based synapses. Figure S3 shows the neuron model and Fibonacci sequence generator. Figure S4 and Figure S5 present the scalable neuromorphic architecture for HCSM and the corresponding programming scheme. Figure S6 and Figure S7 illustrate the open-loop/closed-loop modulation of memristor states. Figures S8-S11 show the encoding result of HCSM for different φ in SPICE simulation.

Synaptic device fabrication and measurement. As shown in Figure S1(a), the memristive synapse is stacked by a typical sandwich structure: TE (top electrode)/Iron Oxide/BE (bottom electrode). The BE, consisting 100 nm TiW and 45 nm Pt, is deposited on a silicon substrate of 535 μm Si and 1000 nm SiO_2. Then a 50 nm iron oxide layer is sputtered at room temperature and a 10 μm $\times$ 10 μm square is patterned under dry etching process. After a 45 nm SiO_2 is deposited, a 0.25 μm^2 nano hole is patterned under dry etching process and the assist of E-beam lithography. At last, the TE consisting 45 nm Pt and 100 nm TiW, is pattered under lift-off process. All the measurements are finished on Keithley instrument. The DC sweeping behaviors are tested under triangle-wave shape DC voltage, linearly increased (decreased) from 0 V to 0.65 V (from -0.65 V to 0 V). The gradual modulation is tested under applied pulse train, with amplitude and width of 1.6 V and 10 μs. After each pulse tuning stage, the resistance is non-disturbingly read at a small DC voltage of 100 mV.

Figure S1(b) presents the well-known I-V hysteresis loops of memristor [4] when triangle-wave-shaped DC voltage sweeps are applied. As opposed to abrupt changes commonly seen in memristive states, the hysteresis loops between every two adjacent sweeping cycles are not overlapped and almost shoulder-to-shoulder. This indicates that the conductance of the memristor can be monotonically and consecutively modulated between intermediate states. Such an extraordinary analog switching property is crucial for the

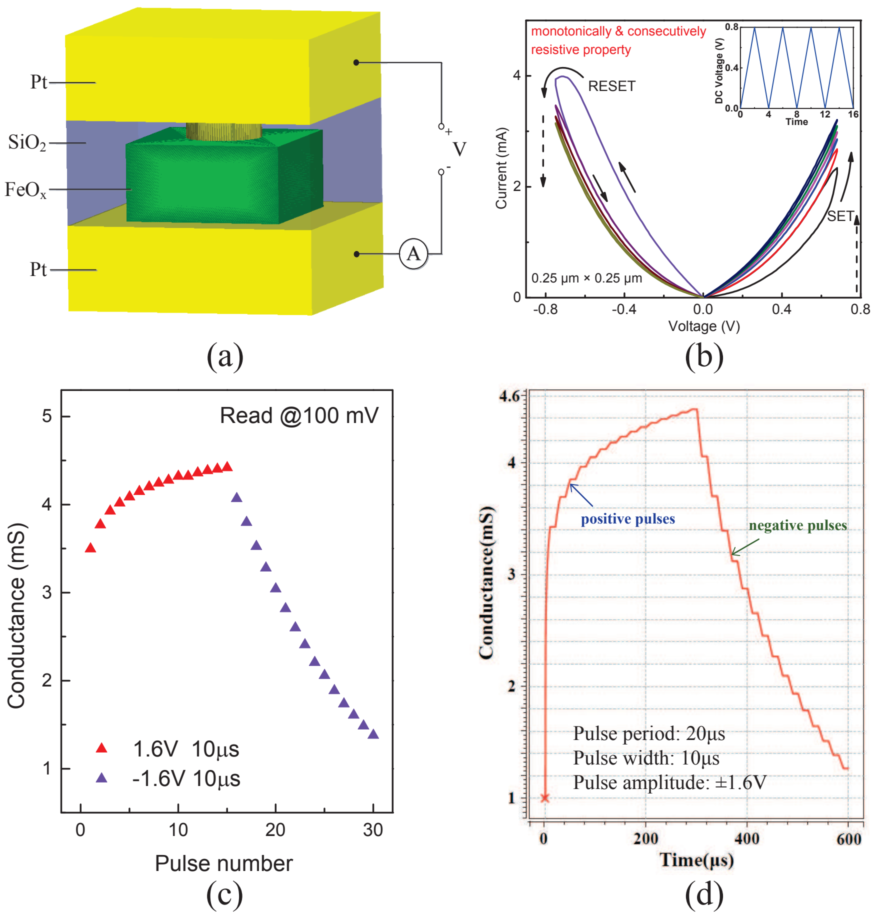

Figure S1: **Artificial synapse based on iron oxide memristor.** **(a)** An illustrative diagram of the FeOx-based memristor device fabricated in this work for synapse emulation. **(b)** A monotonous and consecutive I-V curve, similar to the required analog synaptic behavior, is measured under triangle-shaped DC sweeps. **(c)** The synaptic plasticity is demonstrated by the incremental potentiation (depression) of memristive conductance under consecutive positive (negative) pulses. **(d)** SPICE simulation results of the conductance switching behavior of the iron oxide memristor.

synapse emulation application. To observe the change of synaptic weight, a gradual tuning of the device conductance states is experimentally measured by applying a series of identical voltage pulses. The weight of memristive conductance-based synapse with respect to a series of positive and negative pulses ($+/-1.6\ V, 10\ \mu s$) is measured and shown in Figure S1(c). Clearly, positive pulses incrementally potentiate the weight while negative pulses incrementally depress the weight. The phenomenon corresponds to the short/long-term potentiation (STP/LTP) and the short/long-term depression (STP/LTD) process of synaptic plasticity [5]. In Figure S1(d), simulation results of SPICE model of the iron oxide memristor are provided, which show excellent resemblance with the measurement results in Figure S1(c). It is worth noting that, as strong nonlinearity exists in the modulation process of synaptic weight, the weight will not significantly change if low voltage is applied, whereas it will abruptly start the gradual tuning process

if the voltage amplitude of the applied pulse is higher than its threshold [6]. This is why efficient write and non-disturbing read make it possible to precisely modulate and measure the state of memristor-based neuromorphic networks during training.

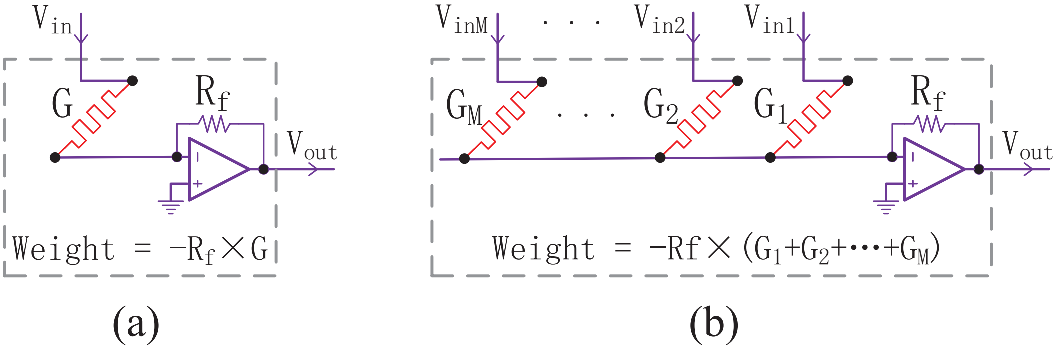

Figure S2: **Synapse structure based on memristor.** **(a)** One-input-one-output structure, the 'single memristor & single amplifier' is equivalent to a dimensionless weight coefficient indicating the signal transmission efficiency from input to output, which bears resemblance to biological synaptic weight. **(b)** Multiple-input-one-output structure, the 'multiple memristors & single amplifier' is able to calculate a multiplication and accumulation (MAC) operation, which is a basic operator for most neural networks. The accumulation function of the amplifier results from the parallel structure of memristors, which is similar to the dendritic integration. Scaling this structure to a 'memristor crossbar & amplifier array', the vector-matrix multiplication (VMM) operation can be easily implemented.

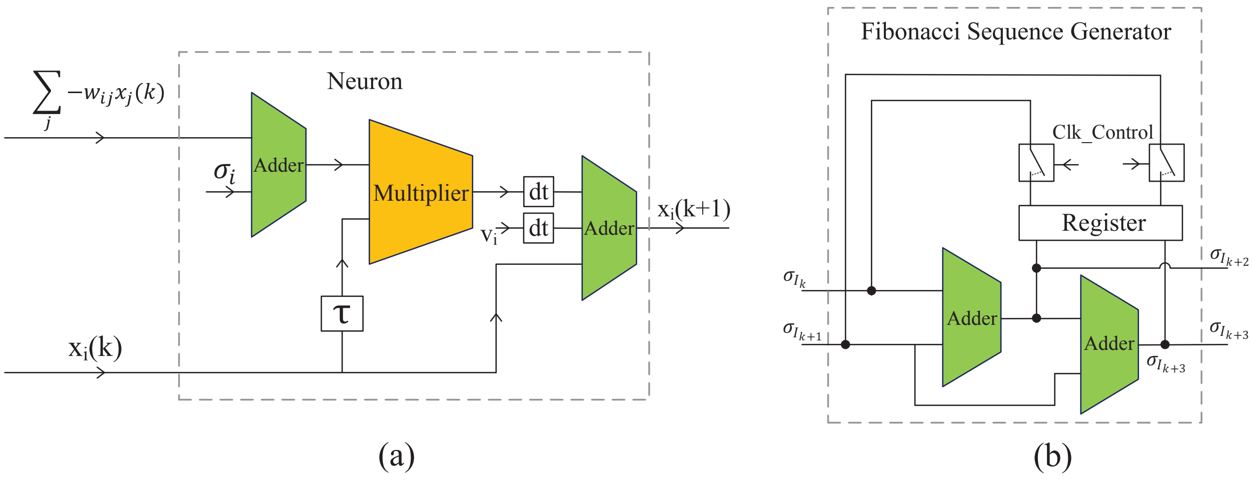

Figure S3: **Schematic illustration of neuron model and Fibonacci sequence generator.** **(a)** An example of neuron to realize the dynamics described in Eq. (6) in the main paper, which is comprised of two adders and one multiplier. It can be observed that the current neuronal activity is generated according to the previous activity and current dendritic inputs. **(b)** Essential Fibonacci sequence generator in neuronal dynamics and weight encoding. By utilizing two adders, Fibonacci sequence can be generated dynamically, and the σ_i and the upper/lower bounds of the synaptic weights can be determined accordingly.

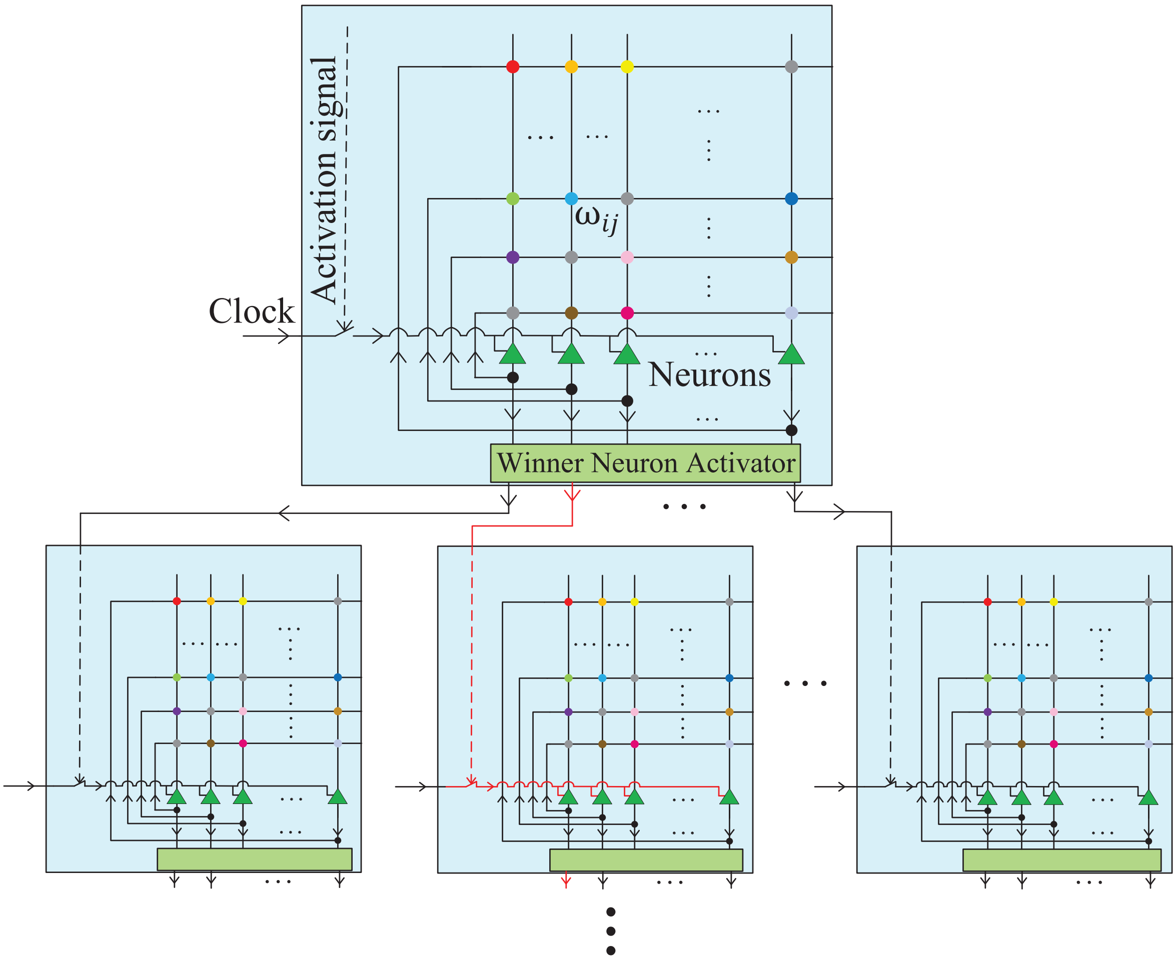

Figure S4: **Illustration of the scalability of hierarchical neuromorphic architecture for HCSM.** Each block has the same structure and function of the single chunk shown in Figure 3. The Winner Neuron Activator in the PC activates its connected CCs in turn based on the current winner neuron. By this hierarchical way, the neuromorphic architecture is scalable to perform the multi-layer model in Figure 2(b).

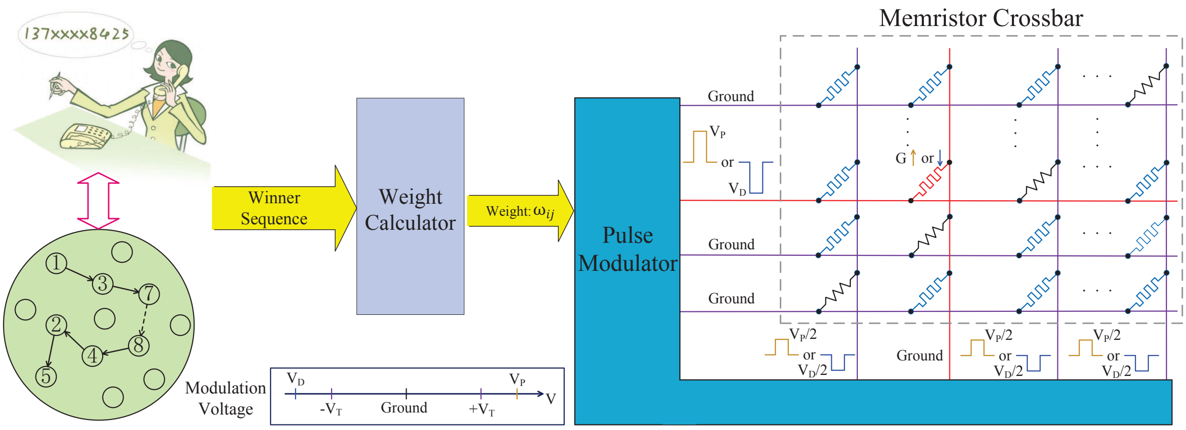

Figure S5: **The programming scheme of the memristor-based HCSM neuromorphic network.** The weight calculator produces a target matrix of synaptic weights, according to the pre-defined winner sequence of a specific memory trace task. Depending on following modulation methods in Figure S6 or S7, the pulse generator block produces corresponding STP/LTP pulse train or STD/LTD pulse train to potentiate or depress the selected synapse. While one synapse is under programming, the others are clamped at their current states with a lower half-selected voltage.

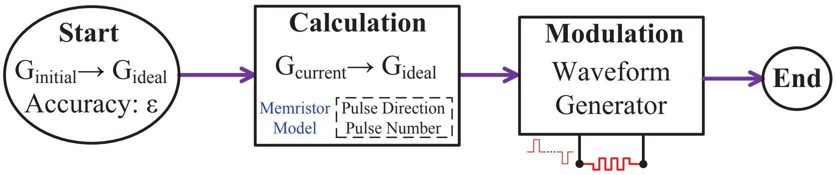

(a)

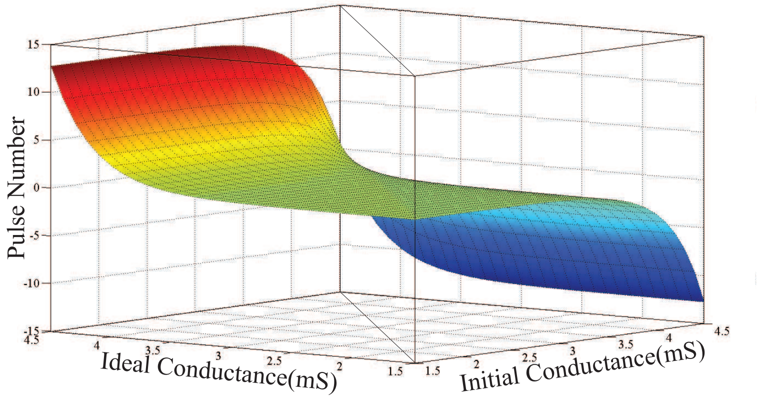

(b)

Figure S6: **Open-loop modulation of memristor states in the pulse generator.** **(a)** Flow diagram of the open-loop modulation scheme, which is adopted to move the initial state of memristor to an ideal state based on the ideal memristor model. **(b)** Illustration of how the Calculation block determines the pulse direction and pulse number. If the initial conductance is smaller than the ideal value, the positive pulse train is applied to potentiate the memristor; if the situation is reversed, the negative pulse train is applied to depress the memristor.

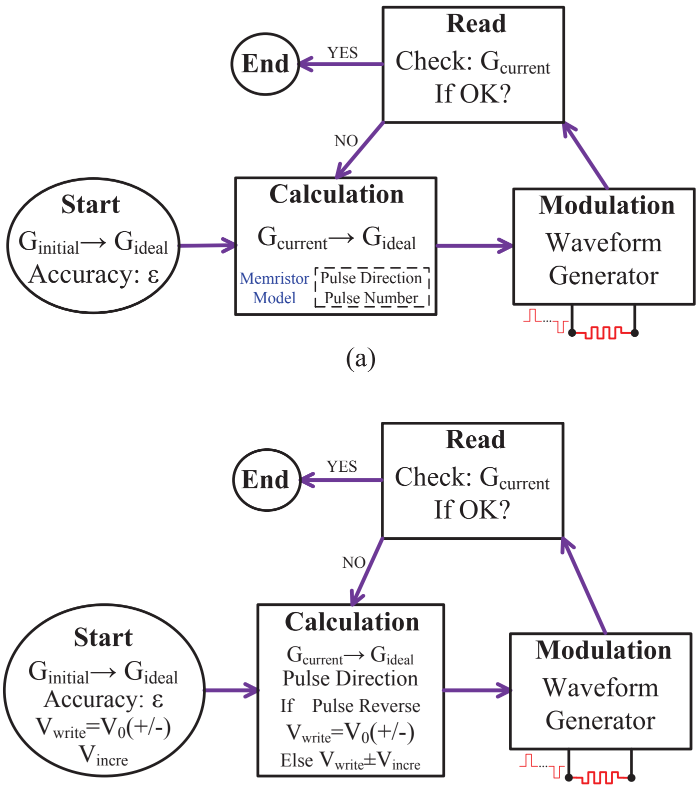

(a)

(b)

Figure S7: **Closed-loop modulation of memristor states in the pulse generator.** **(a)** With the aid of the ideal memristor model, the pulse direction and pulse number can be adjusted dynamically during each modulation cycle. **(b)** A trial-and-error method, the amplitude of pulse is increased gradually due to the lack of ideal memristor model. It is noted that method (b) outperforms method (a) in handling the device variation of memristors, though at a cost of longer modulation time.

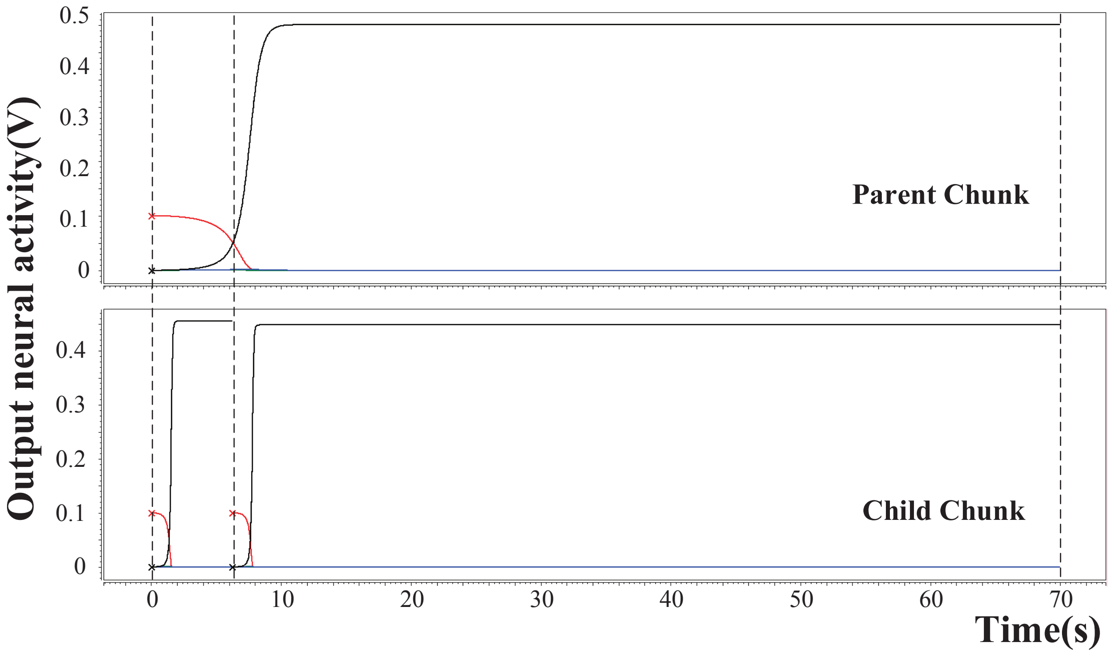

Figure S8: The resulting memory trace of HCSM when $\varphi = 2.0$ in SPICE simulation.

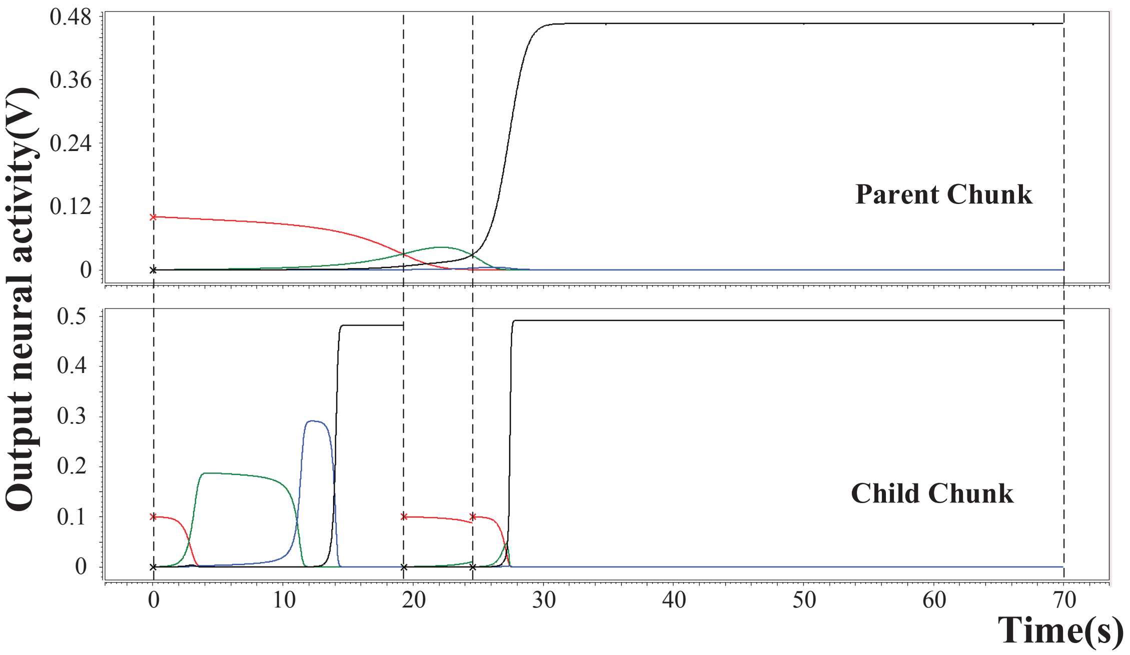

Figure S9: The resulting memory trace of HCSM when $\varphi = 2.4$ in SPICE simulation.

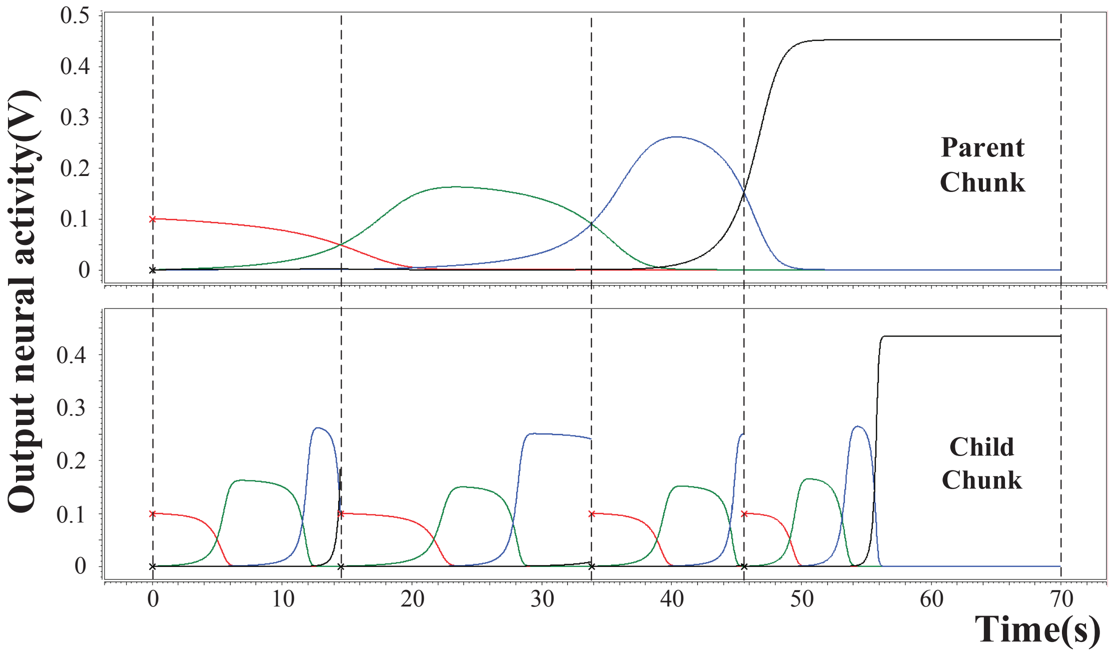

Figure S10: The resulting memory trace of HCSM when $\varphi = 3.0$ in SPICE simulation.

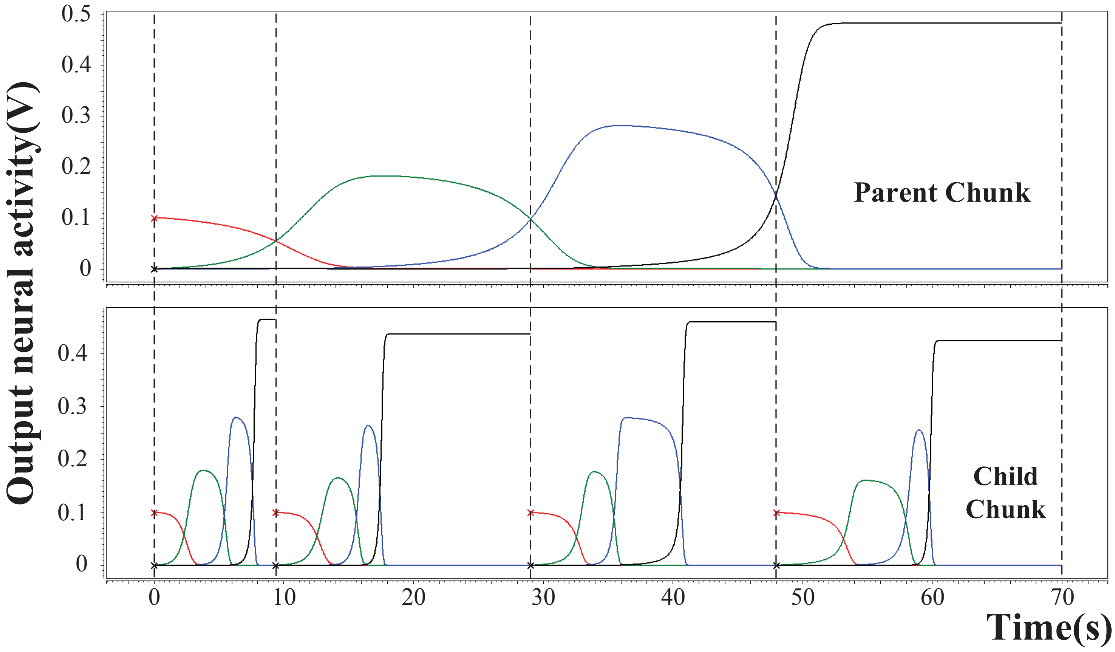

Figure S11: The resulting memory trace of HCSM when $\varphi = 3.6$ in SPICE simulation.

References

[1] Afraimovich, V. S., Zhigulin V. P. & Rabinovich M. I. On the origin of reproducible sequential activity in neural circuits. *Chaos* **14**, 1123-1129 (2004).

[2] Li, G., Ning, N., Ramanathan, K., He, W., Pan, L., & Shi, L. Behind the magical numbers: hierarchical chunking and the human working memory capacity. *Int. J. Neural. Syst.* **23**, 1350019 (2013).

[3] Hahnloser, R. H. R., Seung, H. S. & Slotine, J. J. Permitted and forbidden sets in symmetric threshold-linear tetworks. *Neural Comput.* **15**, 621-638 (2003).

[4] Chua, L. O. Resistance switching memories are memristors. *Appl. Phys. A* **102**, 765-783 (2011).

[5] Bi, G. & Poo, M. Synaptic modifications in cultured hippocampal neurons: dependence on spike timing, synaptic strength, and postsynaptic cell type. *J. Neurosci.* **18**, 10464-10472 (1998).

[6] Jo, S. H., Chang, T., Ebong, I., Bhadviya, B. B., Mazumder, P., & Lu, W. Nanoscale memristor device as synapse in neuromorphic systems. *Nano Lett.* **10**, 1297-1301 (2010).
